# Low-Cost Open-Source Melt Flow Index System for Distributed Recycling and Additive Manufacturing

**DOI:** 10.3390/ma17235966

**Published:** 2024-12-05

**Authors:** Dawei Liu, Aditi Basdeo, Catalina Suescun Gonzalez, Alessia Romani, Hakim Boudaoud, Cécile Nouvel, Fabio A. Cruz Sanchez, Joshua M. Pearce

**Affiliations:** 1 Department of Electrical and Computer Engineering, Western University, London, ON N6A 5B9, Canada; dliu428@uwo.ca (D.L.); aromani2@uwo.ca (A.R.); 2 Department of Chemical and Biochemical Engineering, Western University, London, ON N6A 5B9, Canada; 3 Ivey Business School, Western University, London, ON N6A 5B9, Canada; 4 Équipe de Recherche sur les Processus Innovatifs (EPRI), Université de Lorraine, F-54000 Nancy, France; catalina.suescun-gonzalez@univ-lorraine.fr (C.S.G.);; 5 CNRS, Laboratoire Réactions et Génie des Procédés (LRGP), Université de Lorraine, F-54000 Nancy, France

**Keywords:** material extrusion, material properties, melt flow index, open hardware, polymers, recycling, rheology, thermal properties

## Abstract

The increasing adoption of distributed recycling via additive manufacturing (DRAM) has facilitated the revalorization of materials derived from waste streams for additive manufacturing. Recycled materials frequently contain impurities and mixed polymers, which can degrade their properties over multiple cycles. This degradation, particularly in rheological properties, limits their applicability in 3D printing. Consequently, there is a critical need for a tool that enables the rapid assessment of the flowability of these recycled materials. This study presents the design, development, and manufacturing of an open-source melt flow index (MFI) apparatus. The open-source MFI was validated with tests on virgin polylactic acid pellets, shredded recycled poly(ethylene) terephthalate glycol flakes, and high-density polyethylene/poly(ethylene) terephthalate blends to demonstrate the range of polymer types and recyclability. The proposed MFI tool offers a user-friendly and cost-effective solution for evaluating the flow properties of materials from waste streams, thereby enhancing their viability for additive manufacturing applications.

## 1. Introduction

Due to a wide range of beneficial properties, synthetic polymers generally derived from fossil fuels are in widespread use, as over 390 million metric tons of plastic are produced globally [1]. One property, which has turned out to be something of a curse, is that most plastics have great durability and are resistant to degradation, enabling them to persist as environmental pollutants for hundreds of years [2]. When not properly disposed of, plastics can cause environmental pollution [3], harming wildlife and ecosystems [4], as well as humans [5,6]. Therefore, it is important to manage the waste plastic and recycle it to minimize the harm caused by waste plastic to benefit both the environment and humanity [7]. Unfortunately, only 9% of plastic has been recycled [8]. Of the rest, half is landfilled, 22% is mismanaged, and 19% is burned or incinerated [8]. Of the unrecycled plastics, thermoplastics present a substantial opportunity to profitably utilize available materials, reduce pollution, and redirect waste from landfills. A new approach to developing a circular economy for plastics is being developed in the additive manufacturing (AM) industry. AM technologies create three-dimensional objects by adding material layer upon layer, reproducing geometries from digital 3D models [9,10]. They allow manufacturing components with internal geometries and complex shapes, fostering customization for small batches of parts ranging from prototypes to final products. According to ASTM, AM includes different kinds of processes, such as Fused Filament Fabrication (FFF), Selective Laser Sintering (SLS), Direct Energy Deposition (DED), Powder Bed Fusion (PBF), and Stereolithography (SLA), resulting in a broad range of materials, e.g., plastics, metal alloys, or ceramics [11]. Furthermore, novel materials, such as hydrogels, shape memory polymers, and composites, are increasingly being studied to further enlarge the versatility of this technology [12,13].

Thanks to the wide range of possibilities allowed by AM, the current applications encompass different sectors, ranging from automotive and biomedical to cultural heritage and aerospace fields. For example, SLA and SLS are used for mold and tool manufacturing for the low-volume production of plastic parts, whereas DED and PBF are suitable for metal part production, including gearboxes and valves [10,14]. Prosthesis, dental implants, and assistive aids can be easily customized to match the specific user requirements thanks to AM, and further applications have been studied for tissue engineering, regenerative medicine, and drug delivery systems [13]. The easy and quick replication of parts also facilitated the adoption of AM for educational and preservation purposes, e.g., connection to cultural heritage studies [15]. Moreover, its in-situ use can foster future applications in extreme contexts, such as space exploration and habitat construction [9].

Focusing on AM and environmental sustainability, circular economy within the AM context has been shown to be profitable [16,17] using the process of distributed recycling for additive manufacturing (DRAM) [18,19]. In this model, prosumers (a portmanteau of producing consumers) have a direct economic benefit by offsetting the purchase of plastic products by recycling their waste into custom open-source products [17]. This model is in contrast to traditional centralized recycling models for plastic, which provide no incentive to consumers because of the low value of low density of collecting and transporting post-consumer plastic waste [19]. DRAM plastic converted to AM feedstock will increase in value between 4 and 20 times with the value of bulk commercial plastic pellets (USD 1–5/kg) or commercial filament (~USD 20/kg). With DRAM, greenhouse gas (GHG) emissions from the embodied energy of transportation are minimized for AM materials [20,21]. This result is possible in the most extreme case of consumers manufacturing their own products from their own waste in their own homes [22,23]. Further reductions in GHG emissions are possible when DRAM uses renewable energy like solar photovoltaic technology [24]. Community-level DRAM using a recycling network is also feasible [25,26] and still more efficient than centralized recycling [27]. Overall, DRAM models challenge global value chains already under assault with distributed manufacturing [28].

To facilitate DRAM, open-source (OS) waste plastic extruders called recyclebots [29] have been developed to provide filament feedstock for FFF processes. Recycled waste plastic filaments can be used in any low-cost self-replicating rapid prototype (RepRap)-class [30,31,32] 3D printer. A wide range of recycled plastic has already been shown to be DRAM-compatible, including the following:

- Polylactic acid (PLA) [33,34,35];
- Acrylonitrile butadiene styrene (ABS) [17,36];
- High-density polyethylene (HDPE) [37,38];
- Poly(ethylene) terephthalate (PET) [39,40];
- Linear low-density polyethylene (LLDPE) and low-density polyethylene (LDPE) [41,42];
- Polypropylene (PP) and polystyrene (PS) [38];
- Elastomers [43];
- Carbon-reinforced plastic composites [44];
- Waste wood fiber-reinforced plastics [40,45];
- Fiber-filled composites [46,47].

Furthermore, the filament-making step can be avoided by 3D printing directly from shredded waste in Fused Particle Fabrication (FPF)/Fused Granular Fabrication (FGF) systems [48,49]. Using direct screw-assisted extruders, either single- or twin-screw systems, further expands the range of available DRAM materials [19,50], including polymer blends and multi-material recycling thermoplastics [50,51], multiple-recycled flake feed-stocks [48,52], and bio-based thermoplastics or composites [53,54,55]. Moreover, it improves the economics of DRAM [52,53,56], reducing the cost of producing the material and environmental pollution [57,58].

There are, however, challenges remaining in DRAM, especially focusing on the materials qualification step. For example, different types of polymers require distinct recycling processes, making proper sorting essential [59]. Impurities, contaminants, and the complexities of recycling multi-polymer waste streams pose significant challenges due to incompatibility issues. These impurities affect overall processability, such as flowability and printability, and diminish technical properties, e.g., mechanical and rheological properties. At the same time, incompatibilities increase material heterogeneity, making it difficult to accurately predict changes in properties without experimental work and material characterization techniques. For unknown plastics of different types, rapid characterization methods can help to identify the potential processability of the material to be used in DRAM, reducing the impacts and scraps generated in the initial tuning of the 3D printing setup. One proposed method is the melt flow index (MFI) measurement, which is a good reference for the melt flow behavior of a thermoplastic material, hence its flowability during the 3D printing process [60,61]. MFI, or melt flow rate (MFR), measures the ability of the molten polymer mass to flow through an orifice or die below a heated barrel under a given loading pressure and temperature for a given amount of time [62], usually measured in g/10 min. MFI is a proxy index feature related to the material’s molecular weight [63], which means it can provide information about the material’s viscosity and flowability, allowing a better understanding of its potential behavior in the 3D printer [59]. It should be noted that MFI is measured at a low shear rate compared to the real stress during 3D printing. In general, the higher the MFI is, the lower the viscosity and molecular weight of the tested material, increasing the flow per unit time. This value can be used to determine other properties, e.g., melt volume rate (MVR) and intrinsic viscosity, giving further insight into the rheological behavior of the material [64]. MFI also helps set an initial melt temperature without the need for more complex characterization methods, such as differential scanning calorimetry (DSC). Although other thermal properties also affect printability in FFF/FGF, e.g., crystallinity index [65], the immediacy of MFI measurements and the availability of standardized methods make it an accessible and reliable way to assess the printability of thermoplastics [66]. For DRAM operationalized at the community scale, MFI represents a potential means to quickly screen potential AM feedstock materials [60,67], as the material characteristics could be changed after one or multiple recycling processes due to the thermomechanical degradation of thermoplastics during the recycling and extrusion steps [48,68]. For example, the MFI of ABS will increase after recycling due to degradation, leading to a decrease in its viscosity [69], and hence, its flowability increases. Thus, new 3D printing temperatures or speeds may be applied. In addition, MFI is not only used to assess the behavior of pure thermoplastic materials; it also provides a quick evaluation of the flowability and printability of materials that contain additives, such as compatibilizers [70], chain extenders [71,72], fillers [73], and bio-sourced components [74,75], further expanding its use. This method is especially useful with unknown plastics, such as recycled feedstock or scraps from waste streams, as the experimental values can be compared with previous values from literature and technical data sheets. This practice can help preliminarily assess the kind of material constituting the recycled feedstock, or check the variability of the values to assess the presence of different thermoplastics or impurities.

To rapidly assess the printability of recycled materials, MFI presents a viable and rapid alternative to complex and longer rheological measurements [76,77]. Unfortunately, proprietary MFI systems are still expensive, at around CAD 4700 from Amazon [78] and CAD 2000 from AliExpress [79], and more affordable options are currently missing. With the costs of commercial MFI devices being, in many cases, more expensive than desktop 3D printers themselves, the technique has not been widely used outside of research laboratories. The high costs of MFI devices limit accessibility to resource-constrained settings for which DRAM is perhaps the most promising, because prosumers could trade their labor for high-value products for themselves or to sell. Furthermore, exploiting rapid characterization tools for DRAM could improve the overall quality of the 3D-printed products produced by the prosumers, hence increasing the adoption of this technology and its reliability for product applications, e.g., reducing failures and material scraps from parameter tuning.

To address this issue, this article introduces a low-cost open-source Melt Flow Index er, whose cost is lower than most commercial products, i.e., around CAD 318.15 before tax. First, in Section 2, the basic functioning of an MFI is outlined. In addition, the operation and design of each of the major subassemblies of the OS MFI device are then detailed. The complete bill of materials (BOM) and assembly instructions are provided in Appendix A and B. Next, the method used for the OS MFI device is validated against a gold-standard commercial MFI, which is also described via material tests. In Section 3, the results of using the OS MFI are provided along with an assessment and statistical analysis of some common thermoplastics, including waste plastics for DRAM—virgin PLA pellets, shredded recycled poly(ethylene) terephthalate glycol (PETG) flakes, and recycled HDPE/PET flake blends. The results are compared and discussed in Section 4 in the context of DRAM. Finally, conclusions are drawn in Section 5.

## 2. Materials and Methods

An MFI device is a measuring tool, which can measure the MFR of a thermal-sensitive polymer material. An MFI device normally comprises load weights, a piston, a heated barrel with a die, a cutter system, and a scale. Usually, the procedure of measuring MFR acts by the following steps: first, heat up the barrel to the desired temperature; second, add the polymer sample into the heated barrel and wait for a certain time until the sample melts, i.e., preheat time; third, put the weight on the top of the piston, then after a certain period of time, measure the mass of the extrudate forced to pass through the die. Before and after the tests, the barrel and die should be cleaned to ensure no contamination and the repeatability of the tests, which usually follow ASTM or ISO standards to define the timings, temperatures, weights, and dimensions of the main MFI components [64]. Among the common commercialized MFI systems, some of them are cumbersome to operate and are not automated. For example, the MFI products from MRCLAB need operators to put the weight on the top of the instrument and count down the time [80]. Normally the operator needs to collect the samples and weigh them afterward, such as for many melt flow indexers on AliExpress [79]. To address this issue, the open-source MFI designed in this article was developed to be mostly automated. In the proposed design, the OS MFI can apply loads automatically via the DC motor, cut the extruded material, and weigh it automatically.

To fulfill the functionality of the MFI, the system here described relies on mechanics, electronics, and free and OS software, all of which are available on the Open Source Framework [81]. The BOM for the make, mechanical, and electronical parts are included in Appendix A. The mechanical components are composed of aluminum extrusion profiles, 3D-printed parts, and fasteners. The overall structure is a vertical frame that holds a cylinder, which works as the heated barrel to melt the sample materials. In the electronic part, the instrument is controlled by a Teensy 4.0 board [82], and temperature control, motor, and digital scale systems are integrated into one control system. The whole process will be controlled by the code upload to the teensy board to make it easy to operate and automatic. The cutter on the MFI device can cut the extrusions at set intervals to automatize extrudate collection from the die, and the digital scale can weigh them and record data. Finally, the device designs are meant to be open-source, hence digitally replicable and low in cost. All the bought components used for the hardware design are readily available on the market. Custom parts are designed to be quickly produced with low-cost desktop-size FFF 3D printers and commercial filament feedstock, i.e., PETG and polycarbonate (PC). The structure of the frame is designed to optimize material usage, hence, to be produced with less material and require a limited amount of bought components. For this reason, far less machining is needed than conventional MFI devices, further reducing costs.

### 2.1. Mechanical System

**Figure 1:**
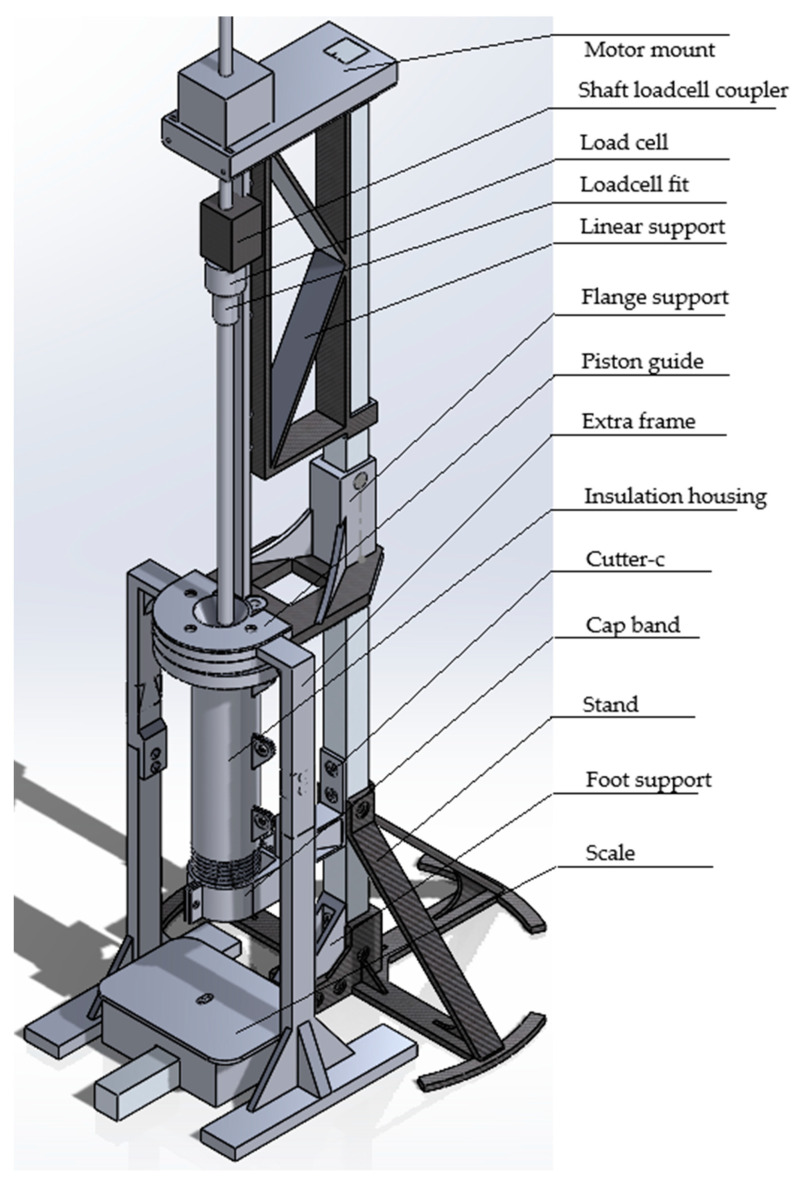
Overall hardware structure of the OS MFI device, highlighting the different parts of the assembly reported in the BOM (Appendix A, from top to bottom): motor mount; shaft loadcell coupler; load cell; loadcell fit; linear support; flange support; piston guide; extra frame; insulation housing; cutter-c; cap band; stand; foot support; and scale.

**Figure 2:**
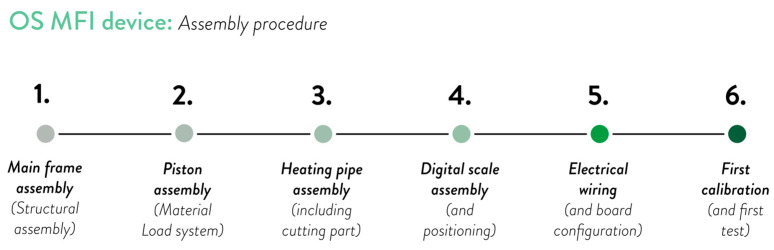
Steps of the assembly procedure followed before using the OS MFI device: (1) main frame assembly; (2) piston assembly; (3) heating pipe assembly; (4) digital scale assembly; (5) electrical wiring; and (6) first calibration.

The design consists of four subassemblies, as follows: frame subassembly, piston subassembly, heating pipe and cap subassembly, and digital scale subassembly. The overall design of the mechanical system of the MFI device is shown in Figure 1, and the BOM is visible in Appendix A.1. The building instructions are reported in Appendix B.1, divided according to the different subassemblies. The whole assembly procedure is resumed in Figure 2, and the CAD files are available in the OSF repository [81].

The framework (frame subassembly, Appendix B.1.1) consists of two 20 × 20 mm aluminum profiles, 75 mm and 30 mm long, respectively, and some 3D-printed parts (Figure 1). This framework connects the different subassemblies together and keeps the whole structure steady. Compared to commercial MFI products, this design uses a stepper motor as the weight on the top of the piston (piston subassembly, Appendix B.1.2). With the linear rail, the shaft of the motor can move up and down on the same track, which helps decrease the vibration on the motor rod. The piston is made of a piston head and a piston rod, and the piston head has a multi-layered structure, which consists of two nuts, one O-ring, and one washer. The O-ring keeps the piston head from leaking. The piston rod is a threaded steel rod, which allows the piston head to screw onto it. There is a button-shaped load cell between the motor shaft and piston rod, which can measure the pressure so that there is a feedback loop for the motor to keep the same pressure. The barrel for heating up samples is a 304 stainless steel pipe, which can be found easily on the market (heating pipe and cap subassembly, Appendix B.1.3). A copper cap with a hole is then added to the barrel to act as the die. A PC insulation housing with heat insulation cotton material covers the whole heated barrel to ensure heat stability. Below it, there is a servo motor equipped with a blade that acts as the cutter for the material samples to be weighted (digital scale subassembly, Appendix B.1.4).

### 2.2. Electronics

**Figure 3:**
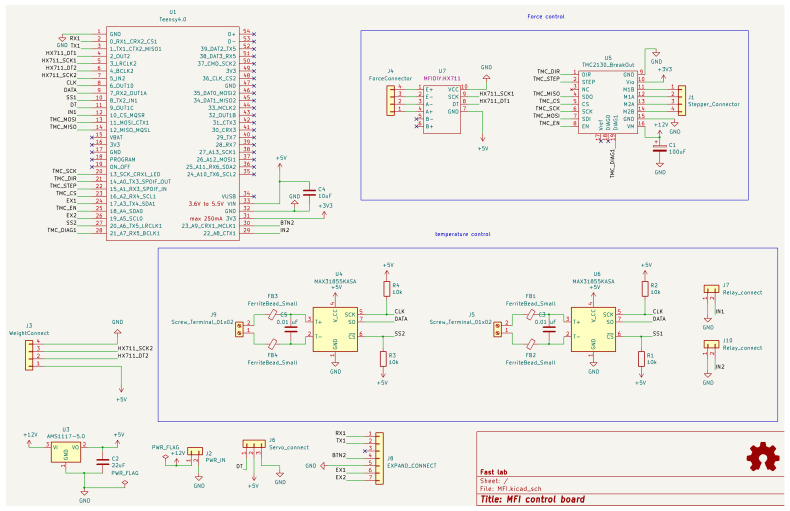
Electronic schematic of the OS MFI device control board (from **top left** to **bottom right**): main control board (**up left**); force control system (**up right**); weight connection system (**left**); temperature control (**right**); low-dropout (LDO) regulator, power connector, servo connector, and connectors (**bottom left**).

To control the system, there is a Teensy 4.0 board that acts as the control board. The control system, as shown in Figure 3, can be divided into two main parts, which are the heating system and the motor force control system. Figure 3 shows each of the main electronic components as well as how they are wired to each other. This includes the OS MFI main control board, force control system, weight connection system, temperature control, low-dropout (LDO) regulator, power connector, servo connector, and the connectors. As mentioned above, there is a feedback loop for the motor. When the force does not reach the desired value, the current of the motor will increase to make the torque output of the motor increase. PID control is applied to the heating control system. All the terms in the PID control system need to be adjusted according to different situations. For example, if the overshot is too high, then it can be improved by reducing the portion term. This system itself runs automatically, but all the input parameters like time, temperature and pressure need to be input manually in the code or firmware. There are two modes in the code, which are auto mode and manual mode. Manual mode is meant for adjusting the position of the piston head and for use in the cleaning procedure after the measuring process. The electronic components are reported in Appendix A.2, whereas the main instructions can be seen in Appendix B.2.

### 2.3. Testing and Validation

#### 2.3.1. Use of the OS MFI Device

**Figure 4:**
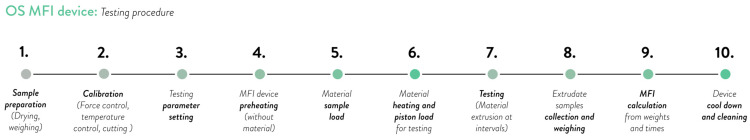
Steps of the test procedure followed using the OS MFI device: (1) sample preparation; (2) calibration; (3) test parameter setting; (4) device preheating; (5) material sample load; (6) heating and piston load; (7) testing; (8) sample collection and weighing; (9) MFI calculation; and (10) device cool-down and cleaning.

The OS MFI device can be used with granulate feedstocks, such as pellets or flakes, and allows the measurement of different thermoplastics, including blends and composites. The operational instructions of the OS MFI device are detailed in Appendix B.3, whereas the whole procedure is resumed in Figure 4. In brief, the material should be dried before measurement to remove residual moisture from the feedstock. After the samples are weighed, the MFI device can be switched on and connected to the laptop through the Teensy board. The operational file (.io extension) can then be opened with Arduino IDE software 1.8.18, and the setup parameters, e.g., temperature, can be defined and uploaded as new firmware onto the MFI board. After the calibration (Section 2.3.2), the user can then use Arduino IDE to input the start commands and monitor the parameters during the heating-up procedure, which should be done without any loaded material. The piston can be moved after the heating-up procedure, and the material can be loaded using command input. The measurement starts when the pressure reaches the value inserted when defining the parameters, and the heated barrel filled with the material sample reaches the temperature set for the test. The piston progressively moves into the heated barrel, pushing the material through the die. The extrudate is then collected after a preset interval, usually 30 s, by automatically cutting it with the cutter blade, helping deposit it onto the scale at each interval. The MFI value can be calculated by collecting each weight measurement reported by the scale. At the end of the measurements, the device can be cooled down and cleaned to remove any residues of material samples into the heated barrel, cap, and die. To improve accuracy and increase the lifespan of the components, regular checking of the extrudate accuracy, consistent room conditions, and cleaning should be ensured, especially with particle-filled materials (Appendix B.3).

#### 2.3.2. Calibration Process

Before testing or validation, the tester needs to be calibrated to ensure precise testing results. Considering the hardware structure reported before (Figure 1), there are three subsystems that need to be calibrated before conducting measurements: (1) the force control system on the piston, (2) the temperature control system of the heated barrel, and (3) the cutter system for the sample collection.

**Figure 5:**
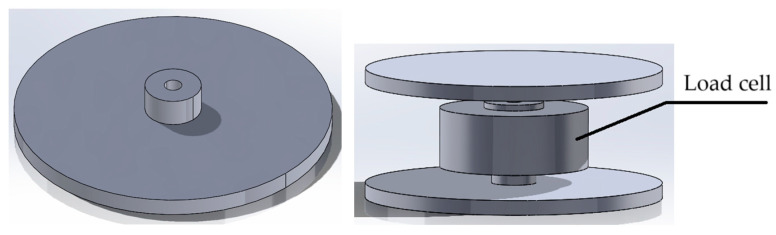
Force control calibration: load cell calibration part to be 3D printed (**left**) [83] and its assembly for the calibration procedure (**right**).

In the force control system, the load cell between the motor rod and the piston needs calibration to ensure the correct load of the material into the heated barrel, controlling the feed rate and flow rate of the sample mass entering the system during the material load. It can be calibrated by using a known weight to be pushed, and a calibration factor can be obtained after this process by calculating the time needed to load the specific amount of material. This factor can then be used to define the required force, and hence, the speed to be set to move the piston through its guide to push the material into the heated barrel for the tests. The detailed operation instructions are included in the calibration code in OSF [81]. To perform the force control calibration correctly, a 3D-printed calibration part should be applied to the OS MFI device (Figure 5 and Appendix A.1, Part 19). The calibration factor may change after the building and first calibration of the device, e.g., multiple measurements or wear, and needs to be checked before testing new samples to ensure repeatability through time. Further details about the methods used for the calibration process can be found in Appendix B.3.

For the temperature control system, the two heating elements of the OS MFI system need to be calibrated to ensure that the actual temperature is the same in the heated barrel and the cap. It can be done using another temperature sensor as a reference to be inserted into the system during the heating-up procedure before material loading, e.g., an additional thermocouple previously calibrated. Another option is to fine-tune the PID parameters in the firmware to match the real performances. This calibration can be done through multiple iterations, comparing the actual temperatures and adjusting the nominal value in the firmware, or by using a reference feedstock for the first measurements to compare with MFI values found in the literature, such as PLA pellets, changing the nominal temperature in the firmware to reach comparable MFI results. The firmware used for the OS MFI device is available in the OSF repository [81]. In the current version, the temperature is stable in the heated barrel of the OS MFI system, but there is a ~5 °C gap between the inside and outside of the cap, requiring an adjustment of the nominal temperature setpoint on the cap from the firmware to reach a consistent actual temperature between the two parts. This fact may be due to different heat loss rates of the parts or insulation issues on the cap, making calibration important to reach stable and precise temperature values before and after loading the material.

The movement of the cutter system can be adjusted in the code in terms of cutting time interval or moving range of the tool, hence by increasing or decreasing the time interval or the movement angle of the stepper motor. The first measurements with benchmark commercial materials can be used to understand the variability of the collected sample mass, increasing or decreasing the moving range of the blade to ensure consistent weights and avoid any loss, e.g., stopping the sample on the blade without reaching the scale. As the position of the blade may affect the cutter performance, it needs to be manually adjusted before the testing process. For instance, it can be adjusted by moving the cutter-fix part until touching the bottom of the cap with the blade, releasing and tightening the nuts to move the cutting subassembly closer or away from the die.

#### 2.3.3. Validation of the OS MFI Device Method

To validate the use of the OS MFI, a series of experiments was designed to compare the measurement results of the OS MFI system with a commercial product available on the market. Compared to the commercial versions, the design of the proposed OS MFI device has some differences, e.g., the diameter and length of the heated barrel, from the specifications in the standardization documents, such as ISO-1133 [84] and ASTM D1238 [85]. The design of experiments follows the principles of a controlled variable experiment, using the OS MFI device during its intended use for the MFI measurements. Except for the difference in testing systems, all other experimental conditions, such as the testing parameters and the materials, are kept consistent. The tests used different materials, e.g., virgin PLA, recycled PET, and recycled HDPE/PET blends. The first feedstock was selected as a benchmark for the comparison, using a commercially available material in the form of pellets. The second feedstock was used to test the device with a common recycled material for 3D printing from 3D-printed parts, using it in the form of shredded flakes. The third material category, plastic blends, was selected to simulate using recycled shredded materials from common goods, another possible source for new feedstocks in the DRAM context. To compare the developed MFI with its commercial counterpart, an Instron CEAST MF20 machine (Instron, Norwood, MA, USA), located at the LRGP laboratory in Nancy, France, was utilized for benchmark MFI measurements following the ASTM D1238 [85] standard.

Materials were obtained from various sources, including virgin pellets, recycled plastic waste, blend combinations with compatibilizers, and shredded 3D printing waste from the laboratory. They were dried at 60 °C for two days before the test. Virgin PLA pellets were supplied by NatureWorks (Savage, MN, USA) with a specific gravity of 1.24 g/cc. Recycled HDPE/PET flake blends were obtained from water bottles coming from the French brand Cristaline and processed at the ERPI laboratory in Nancy, France. This material comprised 10 wt. % HDPE (cap) and 90 wt. % PET (body), hereinafter called rPET90/rHDPE10. The same recycled material was tested with an addition of 10 wt. % styrene-ethylene/butylene-styrene block copolymer (SEBS) G-1652 containing approximately 30 wt. % polystyrene units (rPET90/rHDPE10/10SEBS), kindly donated by Kraton Polymers (Almere, The Netherlands). Additionally, recycled PETG from shredded 3D printing waste at the FAST laboratory was tested. The shredded waste parts were originally fabricated using virgin PETG filaments provided by Polymaker (Shanghai, China). To ensure the reliability and repeatability of the process, the analysis was performed on three samples of approximately 5 g for the commercial and 10 g for the developed OS MFI. The materials were tested at temperatures of 190 °C for virgin PLA, 220 °C, 230 °C, and 240 °C for recycled PETG, and 255 °C for rPET90/rHDPE10 and rPET90/rHDPE10/10SEBS, using a 2.16 kg weight. Preheating times of 300 s and 600 s were set for the commercial and OS MFI devices, respectively. These parameters considered the difference in sample weights between the commercial and OS device versions, i.e., increasing the times for the OS MFI. Although the sample weight influences the heating times, and hence the flowability of the material, e.g., decreasing its viscosity, testing bigger samples in terms of mass and selecting longer preheating times helped ensure proper material preheating in the OS device version. Furthermore, this choice helped track the variation in the MFI values for longer times, allowing a deeper evaluation of the MFI trend and the device performances.

The MFI analyses performed with the OS MFI were used to conduct statistical analysis, evaluating the reliability of the tool in terms of the homogeneity of the measurements, considering some well-known factors influencing MFI measurements, i.e., type of material, measurement and residency time, and test temperature. Three different analyses were conducted to this end: (i) a one-way ANOVA on vPLA, rPETG, and PET/HDPE blends considering the different samples, assessing the variation between the different measurements and hence samples; (ii) a one-way ANOVA on vPLA, rPETG, and PET/HDPE blends considering the measurement time intervals, assessing the variation of the measurements through time; and (iii) a two-way ANOVA with replication on rPETG samples at 220 °C, 230 °C, and 240 °C, assessing the effects of the variation of the test temperature on the measurements. The test conditions were consistent across all the measurements within a specific batch, assuming homogeneity within each group of data, which corresponds to a specific combination of tested material and temperature. In addition, the analysis was conducted by assuming the homogeneity of the materials used for the measurements, either virgin or recycled feedstock. Despite the intrinsic variability of recycled feedstocks, this choice matches with the use of a single material batch for all the measurements of each group, ensuring consistency within the MFI values of each group, i.e., the combination of material and test temperature, the sum of squares (SS), the degrees of freedom (df), the mean squares (MS), the value of the test statistic (F-ratio), and the value of probability (*p*-value) were reported for each statistic ANOVA analysis.

## 3. Results

### 3.1. Comparison Between the Commercial and OS MFI Devices

Table 1 presents the results obtained from virgin PLA, rPET90/rHDPE10, rPET90/rHDPE10/10SEBS, and recycled PETG using both commercial and OS machines. The results were then compared with the data sheets of the virgin materials for PLA and PETG filament, as well as values from the literature on 3D-printed PLA [44,60,86,87], PETG [88,89,90], HDPE, and PET [74,91]. It can be observed that the MFI of PLA assessed with the commercial device corresponds to 6 g/10 min and a relative standard deviation of ~13%, demonstrating both the precision and accuracy of the commercial machine. In contrast, the OS machine exhibited a 15% value decrease compared to the commercial machine despite a better relative standard deviation, i.e., ~5% when the preheating time is 10 min, and a comparable value with ~19% standard deviation when the preheating time is 13.5 min. The results are comparable with the state-of-the-art, which shows significant variations in the MFI values, also influenced by the different feedstock batch and grade, i.e., molecular weight and additives of the specific formulation. For instance, Wang et al. obtained an MFI of ~4.3 and ~11.1 g/10 min at 190 °C and 210 °C [60], whereas Nasir et al. reached ~11 g/10 min at 190 °C [87]. Tian et al. reported values of ~2 g/10 min at 180 °C, which significantly increased at 240 °C [44]. In general, the MFI values of 3D-printed PLA range between 6 and 10 g/10 min [86], confirming the reliability of the results obtained from the commercial and OS MFI devices. In addition, these measurements were obtained by using commercially available pellet feedstock, making virgin PLA a primary benchmark measurement for comparing different MFI machines. This kind of material can also be used as a material for the first calibrations and comparisons when building and setting the device for the first trials, being consistent in terms of material formulation and feedstock granulometry and is widely used in the AM context.

Regarding PETG, the filament datasheet indicated MFI values of 3.9 g/10 min and 10.8 g/10 min at 220 and 240 °C, respectively. The value obtained with the commercial machine is 9 g/10 min at 230 °C, with a relative standard deviation of ~6%. This MFI value is within the range of the commercial virgin filament [92], suggesting that the 3D printing and shredding processes do not significantly impact material degradation. Similarly, the MFI obtained with the OS machine fell between the two values from the datasheet, showing a decrease of ~23% compared to the commercial machine. As for PLA tests with the OS MFI, the values are in line with the state-of-the-art, where results of ~10.5 g/10 min have been reported at 240 °C [88]. To ensure comparability with the datasheet, the tests were also conducted at 240 °C. The MFI recorded from the commercial machine was 14.3 g/10 min, while similar values were found for the OS MFI, with a value of 13.6 g/10 min. This slight increase in MFI may be attributed to the minor degradation of the material during the process. The test conducted at 220 °C yielded values of 6.5 g/10 min and 4.2 g/10 min for the commercial and OS MFI, respectively, with this decrease possibly attributed to inherent variability in the testing process; significant variability in the results was also reported for PETG. For example, Vijayasankar et al. reported an MFI of ~20 g/10 min at the same temperature [89]. Kotomin et al. compared the MFI values of PETG at different temperatures, obtaining ~15 g/10 min at 240 °C and more than 30 g/10 min at 250 °C [90]. For this reason, the values derived from the measurements with the commercial and OS devices can be considered reliable, confirming the validity of the OS machine.

The HDPE/PET blends derived from the plastic bottle waste stream showed MFI values of 34.5 g/10 min without a compatibilizer (rPET90/rHDPE10) and 31.1 g/10 min with a compatibilizer (rPET90/rHDPE10/10SEBS) when tested with the commercial device. Compared with the OS device, it can be observed that MFI is ~15% higher and ~34% lower with the compatibilizer and without the compatibilizer, respectively. Considering the lack of direct comparison from the state-of-the-art, some considerations can be made by checking the MFI values reported for HDPE and PET. Previous works obtained MFI values ranging between 3.5 and 40 g/10 min for PET, showing a significant variability given by the pre-heating conditions [91,93,94]. HDPE MFI values from the literature also exhibit the same trend [63]. Nevertheless, the results from the commercial and OS MFI devices are comparable, indicating the reliability of the OS machine in testing novel material compositions and plastic blends.

**Table 1:** MFI test parameters and results (mean values and standard deviations) of virgin PLA (vPLA), recycled PETG (rPETG), rPET90/rHDPE10, and rPET90/rHDPE10/SEBS10 obtained with the commercial and OS MFI devices.

Material	Weight (g)		Preheat Time (s)		Temperature (°C)	MFI (g/10 min)		
	Commercial	OS	Commercial	OS		Datasheet	Commercial	OS
vPLA	5	10	300	600	190	6.0 [95]	6.0 ± 0.8	5.1 ± 0.3
	5	10	300	810	190	6.0	6.0 ± 0.8	6.0 ± 1.2
rPETG	5	10	300	600	240	10.8 [92]	14.3 ± 1.5	13.6 ± 2.7
	5	10	300	600	230	-	9.0 ± 0.6	6.9 ± 0.9
	5	10	300	600	220	3.9	6.5 ± 0.8	4.2 ± 0.5
rPET90/rHDPE10	5	10	300	600	255	-	34.5 ± 4.3	18.3 ± 6.0
rPET90/rHDPE10/SEBS10	5	10	300	600	255	-	31.1 ± 4.3	36.9 ± 15.4

**Figure 6:**
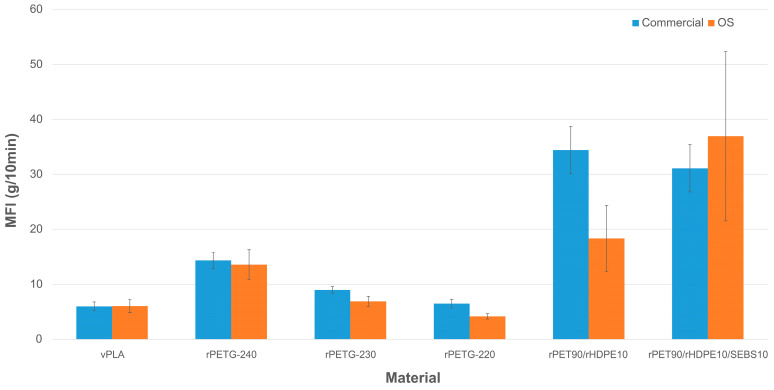
Results of the MFI measurements (from left to right) obtained with the commercial equipment (left side bars, blue) and OS MFI devices (right side bars, orange): (1) virgin PLA (vPLA); (2) recycled PETG (rPETG) at 240 °C, (3) 230 °C, and (4) 220 °C; (5) rPET90/rHDPE10; and (6) and rPET90/rHDPE10/SEBS10.

Overall, as shown in Figure 6, both systems showed similar measurement precision and were capable of stable operation within a standard deviation of ~5–20%. There are still some differences in the values obtained from the two MFI devices in three cases out of seven, i.e., rPETG-230, rPETG-220 and rPET90/rHDPE10, as suggested by the absence of overlap between the error bars. This discrepancy can be due to possible errors in the calibration, as well as the variability of the feedstock itself. According to the data, higher variations were found by testing recycled plastic feedstock, whose properties are significantly different according to the specific batch recycling processing steps and conditions as waste. Although more precise calibration is required, the results indicate that the OS MFI system can measure the MFI of different materials with precision comparable to commercial MFI devices. The data obtained from the OS MFI system can be useful to observe and make relative comparisons of different melt materials or temperatures, outlining the overall behaviors of each processed feedstock. Additionally, the study has demonstrated the use of the OS MFI with different thermoplastic materials, either virgin or recycled. Compared to the PLA test data, there is a larger difference between the weights of extrudates made of PETG and HDPE/PET blends. This phenomenon could be due to the substantial differences in the shape and size of the shredded PETG and HDPE/PET blends, e.g., different granulometries and dimensional ratios between pellets and shredded flakes, leading to the formation of voids during extrusion, thereby affecting the measurement results and increasing their variability [84,96]. For this reason, a first calibration with virgin PLA pellets can help assess the sample mass to be used for the tests, especially during the first calibrations after building.

Considering the application and use of the OS MFI device, the results in Table 2 and Figure 6 give an overall idea of the temperature range to be used with the tested materials. Despite the existing differences between the two devices, the OS MFI tool can give a first assessment of the flowability of the tested material, either in the form of pellets or flakes (Table 2). For instance, testing the same recycled material at different temperatures, as done for rPETG, can help us understand the most suitable temperature for its manufacturing. Focusing on FFF and FGF 3D printing, rPETG should be processed at ~230 °C to reach good flowability, avoiding further thermal degradation from higher processing temperatures (~240 °C) or the too-high viscosity of the extrudate passing through the nozzle (~220 °C). As for rPET/HDPE blends, this approach can also help understand the heterogeneity of unknown recycled materials thanks to the lower accuracy given by the test, represented by the error bar. Although it is a qualitative evaluation, it may support preliminary decisions on using unknown recycled feedstock in real contexts. Another application of the OS MFI is to use it to understand the behavior of the tested material after specific pre-heating times to qualitatively assess the change in the flowability of a 3D-printable feedstock through time, e.g., simulating the different residency times of the feedstock into the 3D printer hotend. This secondary use of the MFI can help us understand the maximum preheating time achievable by a material before 3D printing without affecting its optimal flowability, e.g., during the printing bed preheating.

This overall approach also shows potential applicability for other materials, e.g., fiber-reinforced and particle-filled thermoplastics, allowing the study of new formulations by comparing matrix flowability with different reinforced or filled compositions. In the DRAM context, this option can be used to explore the use of partially or fully recycled composite materials, e.g., containing recycled matrixes or biomass scraps and byproducts. Lastly, it can be exploited for other manufacturing processes used for recycled plastic forming, such as compression molding or thermoforming, further enlarging the range of uses of the OS MFI device.

### 3.2. Validation of the OS MFI Device

**Figure 7:**
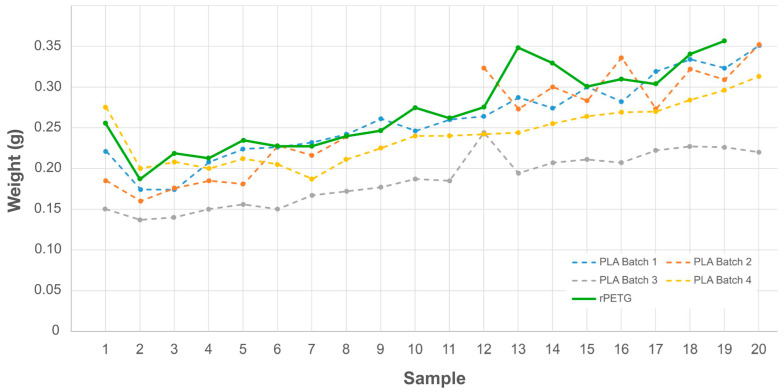
Results of the virgin PLA measurement with the OS MFI device applied to four batches of samples (dot lines, a different batch measurement for each color) compared to rPETG measurement (straight line, green), where each batch corresponds to one measurement. Each sample, indicated by the different dots, was collected after a progressive interval of 30 s during the specific MFI measurement, which means 1 = 30 s, 10 = 5 min, and 20 = 10 min.

After comparing measurements from a commercial MFI device, further analysis was conducted to validate the results from the measurements performed with the OS MFI. According to the results, only the last ten sets of data should be accepted when dealing with data from the OS MFI device. As shown in Figure 7, there is a clear increasing trend of extrusion weight through time for PLA, and all the tested samples show a similar trend when using the OS MFI device. This trend was also confirmed by the rPETG measurements, included in Figure 7 as a comparison. However, the results for commercial equipment do not show this trend. One factor that can cause this phenomenon is the difference between the real temperature inside the die and the nominal one at the beginning of measurement. It may be due to insufficient insulation on the bottom of the cap, which can cause heat loss and less thermal control. The difference in materials between the cap and the heated barrel can also lead to differences in heat transfer, requiring a calibration step of the temperature control system (Section 2.3.1). This fact can be influenced by the device hardware setup and the change in properties of the tested material over time. On the one hand, temperature differences between the tool parts can affect the first part of the test. This fact leads to lower values for the first tested samples, requiring more time for complete temperature stabilization of the material in the barrel and cap. On the other hand, higher residency times of the material in the heated barrel can lead to thermal degradation, hence in lower viscosities and an increase in the MFI over time. A preload process may be needed before the test to yield a more precise result. For example, the first few samples flowing from the die can be discarded as part of the preload process, and the number should be determined through preliminary tests with specific materials. Due to a higher sample mess used in the OS MFI, the whole testing process may take a longer time, which can increase material degradation and affect the results.

**Figure 8:**
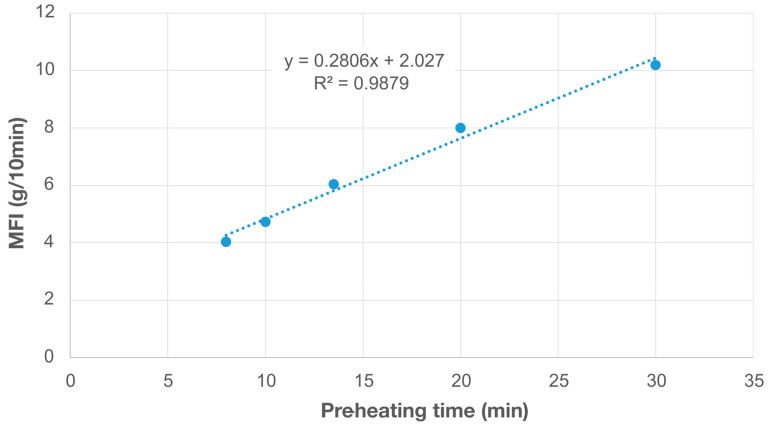
MFI values of virgin PLA changing through different pre-heating times from the tests with the OS MFI device, i.e., 8, 10, 13.5, 20, and 30 min; 8 min corresponds to the minimum preheating time, which means the complete melting of the tested material.

Multiple experiments were performed to verify this phenomenon through OS MFI and to define the correct pre-heating timings. As shown in Figure 8, the material is fully heated within ~8 min. The results show a clear linear increasing trend with the increase in pre-heating time. Therefore, it is important to keep a constant sample weight throughout the tests. Moreover, according to the standard file ISO 1133 [84], material in flake or powder shapes should undergo preprocess, i.e., a pressing process with a vacuum pressing tool to the evacuate air in the samples. This step will reduce the degradation of material caused by oxygen. This fact also explains the severe degradation caused by the rPET90/rHDPE10 measurements, as reported in Table 1 and Figure 6.

The measurements conducted with the OS MFI device were used to evaluate the reliability of the tool through statistical analysis, i.e., one-way ANOVA on vPLA, rPETG, and PET/HDPE blends considering the different samples, one-way ANOVA on vPLA considering the measurement time intervals, and two-way ANOVA with replication on rPETG samples tested at 220 °C, 230 °C, and 240 °C. The analysis considered well-known factors affecting MFI, such as type of material, test time (interval and residency time), and temperature. Moreover, it assumes the homogeneity of the testing conditions within each batch of tested material, either virgin or recycled, focusing on the influence of the device. Despite the possible heterogeneity between different batches of the same recycled feedstocks compared to virgin materials, this choice helps assess the influence of the tool itself on the measurements, e.g., evaluating the statistical difference in changing one of the aforementioned factors or, on the contrary, obtaining results without statistical differences despite their variation.

The first statistical analysis considered the variation between the different samples tested with the OS MFI, hence the influence of the different measurements on the values. According to the one-way ANOVA performed on the material samples (Table 3), the f-values are higher for virgin PLA and recycled PETG, whereas lower F-ratios were found for the two rPET/rHDPE blends. The results indicate a statistical variation of the measurements only in the first two tested materials, vPLA and rPETG (*p* ≤ 0.05), showing a non-significant difference in the PET/HDPE blend samples, e.g., from normal experimental error or variability in the recycled feedstock granulometry and heterogeneity in the same material batch. This fact further supports the reasons mentioned above behind the measurement variation, such as calibration and temperature gradients within the OS MFI device given by the different components. The variations can also be due to differences within the same batch of material, often found in recycled feedstocks, e.g., shape size and dimension, degradation, or crystallinity degree. This fact can be mitigated by improving the granulometry consistency of the tested feedstock, e.g., multiple shredding, sieving, controlled blend mixing, or pellet making. Despite the variation encountered within the samples considered for the first statistical analysis, the influence of the measurement time intervals can also affect the homogeneity of the results, as visible in Figure 7. Table 4 shows the results from the one-way ANOVA analysis, dividing the measurements according to the specific time interval, i.e., every 30 s. In this case, only the vPLA samples show a statistically significant difference, reaching a *p*-value less than 0.05 and a higher f-value, given by the lower variability of the feedstock shape and granulometry in the same material batch. In other words, a homogeneity assumption is not reached for vPLA when considering time intervals, indicating its influence on MFI values. According to this result, the testing procedure should consider specific time intervals, ensuring their consistency when comparing different batches. Some preliminary testing can be used to define the correct interval for each specific material, and the actual measurements can be carried out accordingly. Lastly, a two-way ANOVA analysis with replication was performed to check the influence of the different test temperatures on a specific material, rPETG, whose influence on the MFI measurement is well-known in the literature [50,63]. According to Table 5, only the highest test temperature, 240 °C, influenced the results from a statistical point of view (*p* ≤ 0.05 and high f-value). On the contrary, the samples tested at 230 °C and 220 °C do not show results with a statistically significant difference, meaning that the variability of the results can be due to other factors. Accordingly, the degradation in the heated barrel and the die can lead to significant differences in the measured MFI values when overestimating the test temperature. For this reason, the optimal test temperature should be set through multiple iterations, starting from lower values and progressively increasing the temperature before its variation reaches no statistically significant measurements, i.e., *p*-values higher than 0.05 when analyzing the data.

Despite the variability due to the single measurements, the results show the importance of proper calibration procedures and measurement accuracy to improving the reliability of the OS MFI device, e.g., with a commercially available feedstock, as well as the need for precise measurement intervals to achieve accurate MFI values with the proposed tool. Overall, the statistical information shows that the OS MFI can be used in place of the commercial counterpart for DRAM applications. In this context, increasing the number of tests can help improve the reliability of the results, especially for novel or unknown recycled material feedstocks. Another option to improve the reliability of the results is to test different batches of samples by progressively changing the testing temperature and assessing the presence or lack of statistical differences in the results. This last option can also be pursued by conducting a preliminary test on a well-known virgin available feedstock, using it as a benchmark. Lastly, improving the granulometry consistency of the feedstock can help reduce the statistical difference in the results, e.g., through multiple shredding or using pelletizers, getting closer to the virgin feedstock in terms of homogeneity.

## 4. Discussion

### 4.1. Applications and Relevance to the DRAM Context

At the community scale, developing a distributed recycling process required the whole value chain to be adequate, from waste to product. In that sense, qualifying the plastic waste stock in a quick and efficient manner represents a challenge for the implementation of this process in a particular territory. The melt flow index (MFI) measurement represents a potential means to resolve this issue within different real-world contexts [97]. For instance, this tool can be replicated and used in community-led scenarios, e.g., fab labs and makerspaces, or community recycling initiatives, such as Precious Plastic [98]. Furthermore, its easy replication can be exploited for educational purposes in practical workshops and courses led in fab labs, makerspaces, and schools, providing the basic principles connected to parameter optimization. In addition, small-scale industrial applications, e.g., 3D printing studios and services, can use OS MFI systems to conduct preliminary tests during the process setup of new material feedstocks. This device can help them to quickly assess recycled materials to be further tested and used for product fabrication. Finally, the device can be used in resource-constrained settings, either labs or community-led spaces, thanks to the use of low-cost electronics, other purchased components, and the use of desktop-size low-cost FFF 3D printers to fabricate parts (Appendix A). On the one hand, using commonly available purchased components allows its replication in different socio-cultural and geographical contexts, e.g., remote areas or spaces with limited budget availability, reducing its costs compared to the commercial versions. Small-scale FFF can simplify the production of the custom parts or their modification according to the available purchased components, e.g., when there are shortages or a limited availability of specific parts.

The OS MFI device developed in this research offers an accessible and low-cost way to rapidly assess the flowability and printability of recycled thermoplastic materials and blends. To validate the accuracy of the OS machine, a comparison was conducted with a commercial MFI device and the state-of-the-art. The results show differences between both the OS MFI system and commercial devices compared to the datasheet and the literature. The measurement of the MFI can be significantly affected by the feedstock grade, e.g., molecular weight and specific additives, but also by different parameters such as temperature, load weight, the diameter of the die, the diameter of the barrel, and their cleanness [50,63], as well as the heat transfer conditions in the whole device itself. For instance, increasing the testing temperature of 10 °C can lead to a two- or three-fold increase in the MFI values, such as for PETG feedstock [90]. Moreover, as shown in the results part (Section 3), the pre-heating conditions can also significantly modify the results, e.g., with differences of one order of magnitude [91]. Despite equal temperatures and load weights, the diameters of the barrel and die might impact the measurements, as well as the properties of the materials used for these components, e.g., different heat capacities and specific heats [60]. Changes in these factors reasonably explain the differences in the test results between OS and commercial MFI devices, as well as the results of the statistical analysis on the OS MFI tool. Likewise, inconsistencies can be found regarding the MFI test results in the literature, even for similar materials used with variability in grade. For instance, Singh et al. reported an increase in the MFI of recycled ABS [55], whereas other works demonstrated a decrease in the MFI of the same material [76,77]. Again, these contradictory results highlight the variability in MFI values, which may be influenced by differences in experimental conditions, material processing, measurement techniques, or the measuring equipment used in these studies, as well as different feedstock grades and tested batches. Furthermore, according to Rides et al., MFI measurements show larger differences between different laboratories [64], confirming the variability in the equipment conditions, environment, e.g., room humidity, and setup, especially for polar and relatively polar materials. Nevertheless, using the developed MFI tool can support a quick qualitative identification of the recycled feedstock without additional characterization techniques by providing (i) a first assessment of their flowability in case of less-contaminated feedstock; (ii) a range of processing temperatures if the material has impurities, indicated by the lower accuracy of the measurements; or (iii) an intermediate processing temperature if the feedstock contains different homogeneously-mixed thermoplastics. At the same time, the OS MFI device can help assess the potential use of the tested feedstock, avoiding the use of materials with unsuitable MFI values for the specific manufacturing process or significant degradation and chain-length reduction after multiple recycling. A further option to increase its reliability is to adopt a commercially available virgin material feedstock to be used as a benchmark during the calibrations, e.g., PLA pellets. As pellets can sometimes be difficult to source, a commercial PLA filament can be easily pelletized with an open-source pelletizer [99]. In this way, the measurements can be directly compared with the state-of-the-art and the datasheet, and the device can be tuned to reach comparable results. This procedure can be then used before testing new materials, considering the PLA measurements as a preliminary step to reach more accurate results. This tool can, therefore, simplify a first assessment of recycled feedstocks while reducing the costs and time connected to material identification and characterization equipment.

Providing the whole code for the OS MFI could help overcome this challenge, ensuring that any research lab, community makerspace, or other DRAM facility could use nearly identical systems. Compared to standard commercial MFI devices, the OS MFI developed in this study utilizes a DC motor to apply the load weight, which can impact the measurements, as load weight is a critical parameter. From a user perspective, this feature can simplify the operation and enhance the safety of the measurement process, making it more accessible and repeatable in DRAM contexts. It is important to highlight that MFI is a valuable tool for the rapid assessment of the flowability and potential printability of materials. For more detailed and scientifically rigorous results, however, comprehensive rheological analysis using rheometers should be conducted even when using commercial MFI devices. These tests can help better understand the behavior of the molten material under high shearing, such as in 3D printer extruders. Furthermore, MFI can not only be used in DRAM contexts or recycling activities, but also in laboratory environments [100]. This choice can help make research more affordable and reliable because this characterization can support the fine-tuning of 3D printing parameters with novel material formulations, helping to ensure extrudate consistency and, hence, improving the technical properties of the final parts, e.g., achieving better mechanical properties by reducing voids and defects from extrusion inconsistencies. In addition, this MFI device can potentially be used for the fine-tuning of different manufacturing processes available in research and makerspaces contexts, such as thermoforming or compression molding, increasing the relevance of this tool.

### 4.2. Limitations and Future Work

There are limitations of the OS MFI device that need future work, also highlighted by the statistical analysis. Currently, cleaning procedures are labor-intensive. The cap needs to be disassembled to clean it from the inside, requiring the disconnection of the band heater and thermocouple, which might be damaged after several cleaning cycles and affect the overall accuracy. Creating or identifying specialized cleaning tools to simplify the process could reduce the difficulty of cleaning and reduce the need to disassemble several parts. Another option can be modifying the design of some parts of the assembly to avoid the removal of the heater and thermocouple during cleaning, speeding up the process and limiting damage to wirings and hardware parts. The cleaning and assembly procedures can also be improved to simplify the procedure and increase the device accuracy and lifespan by (i) improving the assembly of the cap to ensure its accurate positioning after several release and tightening rounds, (ii) simplifying the disassembly of the die for replacement, e.g., after several uses or testing composite materials filled with abrasive particles, and (iii) creating an ad-hoc procedure in the Arduino IDE to automatize the input commands used during cleaning. Furthermore, the temperature control system could be improved in accuracy, as it currently shows variations of ~2 °C. Although there are two heating systems, the nichrome wire and the band heater, temperature differences still exist in the MFI device, leading to different MFI values according to the specific pre-heating times (Figure 5). For example, the temperature in the die on the cap could be lower than in other parts, e.g., 170 °C instead of 190 °C, affecting its accuracy and providing results that should be adjusted when using the tested material. The implementation of automated temperature control can be beneficial in adjusting the difference between the heated barrel and the cap, using the heating systems to compensate for the temperature gradient. Future experiments could also consider the thread of the cap. Due to the threaded fit of the cap and the pipe (National Pipe Taper, NPT), the cap cannot thread all the way up. This constraint leaves a part of the thread exposed, and it can affect the shear force when extruding, potentially influencing the beginning of the measurements. This thread type, however, helps prevent leakage, increasing accuracy and limiting the shear force effect. Future versions of the OS MFI device can include an alternative threaded fit to reach a compromise between temperature accuracy and material leakage.

To achieve better accuracy, some improvements can also be made to the device. According to the previous test at 190 °C, the position of the band heater significantly modifies the actual temperature in the die, reducing the overall accuracy. If the position of the band heater is up, roughly where the bottom of the cap can be exposed, the temperature in the die will be 170 °C, while if the position of the band heater is down, roughly where the cap can be completely covered, the temperature will be 182 °C. This test shows that the relative position of the band heater and cap affects the temperature inside the die, showing a significant improvement after fixing the position of the band heater and the cap, e.g., after disassembly, maintenance, and cleaning. These results indicate that the stability of the temperature is a key factor in getting a more accurate MFI measurement, reducing the experimental error during tests. Hence, further accuracy improvements to temperature control might be made by (i) reinforcing the insulation layer and improving the connection between the different parts to be heated and cleaned after the tests, e.g., redesigning the band heater assembly or changing the connection type; (ii) having a more advanced temperature control system, including improved insulation and temperature sensors, to monitor the temperature gradient within the whole heated barrel and the path followed by the tested material before reaching the die; building an enclosure for the OS MFI system to ensure a controlled environment, e.g., external temperature and humidity; (iv) advanced temperature control algorithms to make real-time modifications of the nominal temperature; or (v) automated temperature control systems according to the recycled feedstock or its available information, such as previous product application, recycling process conditions, or granulate shape, type, and dimensions. The accuracy of the digital scale and its interaction with the cutter system can also impact results. In the current OS MFI device, the weight is unevenly distributed on the scale plate, leading to potential measurement errors of the extrudate, e.g., for its shape after cutting. Improvements in scale and cutter design can, therefore, increase accuracy, e.g., designing a sample-collecting system to ensure consistent extrudate shapes for each weighted sample, or replacing the scale and connecting it to the tool board to store and analyze data from the measurements. A better motor control system can also be considered in future versions of the OS MFI device. Current motor control systems are generally accurate and can introduce some errors in the pressure, hence in the extrudate quantity to be weighted at each measurement. Therefore, using more advanced motor control algorithms [101] can make the pressure more accurate, resulting in more consistent samples in terms of extruded mass. Finally, future work could include a Graphical User Interface (GUI) to make the system easier to operate and guide data interpretation and analysis, especially for non-expert users or DRAM contexts. For instance, the GUI could: (i) avoid the use of a laptop with Arduino IDE and command inputs; (ii) suggest a range of potential temperatures according to the first MFI measurements; (iii) include the MFI value calculation as an automatic feature after measurements; (iv) guide the user in the maintenance and cleaning procedures of the device by providing a step-by-step procedure; (v) provide some presets for the most recycled feedstocks for AM, e.g., plastic bottles or 3D-printed failed parts with common filaments; and (vi) collect and save custom settings defined by the user for future measurements of similar feedstocks or benchmark comparisons.

## 5. Conclusions

To address the current issues with MFI systems, a low-cost OS MFI device was developed. MFI measurements of virgin PLA pellets, recycled PETG flakes, and HDPE/PET blends were carried out using the developed device, showing good measurement precision with a ~5–20% standard deviation. Compared to its commercial counterpart, it shows a ~5–23% decrease in PLA and PETG measurements, which reflects a similar trend. The OS MFI can offer a valuable characterization tool for easily sorting different material feedstocks, determining their suitability for FFF/FGF 3D printing, and identifying the optimal 3D printing temperature. This study demonstrated the development and use of an OS MFI with performance comparable to its commercial counterparts. The validation test reported similar MFI values for both the commercial and OS MFI devices when using common 3D printing feedstock, i.e., virgin PLA and recycled PETG, and a new polymer blend formulation from plastic waste, such as HDPE/PET blends. The statistical analysis confirmed the importance of proper calibration, time interval, and temperature settings to achieve accurate results with the OS MFI device. Some challenges remain, however; particularly in achieving consistent temperature along the heated barrel and die.

Future machine versions, therefore, should improve the temperature control system and connection between the cap (die) and heated barrel, optimizing the heat transfer in the whole equipment, e.g., improving insulation or changing part of the buy components selected for the current version. For instance, the temperature control system can be modified or changed to ensure more accurate temperatures over time, improving the affordability of the tool. Another future modification can involve the maintenance and cleaning steps, making the assembly quick to disassemble in case of spare parts replacement. As an example, the assembly of the insulation and cap heater can be adjusted to avoid its removal during cleaning, simplifying and quickening the procedure. Some custom 3D-printed parts can also be modified or redesigned to improve material usage and simplify the assembly, e.g., avoiding supports on the stand part or studying part consolidation. To enhance user-friendliness, future work can develop a graphical user interface, guided testing and analysis procedures, and accessible tutorials that enable the adjustment of test parameters, such as pre-heating, weight load speed, or automated cutting timings. Finally, future work should: (i) explore the use of the OS MFI device with additional recycled feedstocks, e.g., different recycled thermoplastics, waste sources, or particle types and granulometries; (ii) deepen the ANOVA statistical analysis, e.g., studying the variability between different batches of the same recycled material; and (iii) consider other test variables affecting the results, such as humidity or multiple recycled feedstocks.

The MFI devices in the market are currently expensive. An OS MFI device can make the recycling process easier and more efficient, becoming a valuable option in different contexts, such as DRAM and laboratory environments. A more affordable and accessible version of MFI devices can help enable DRAM to become more widespread and trustable in terms of material manufacturing processing, encouraging the use of recycled feedstock for 3D printing and conventional manufacturing processes.

## Figures and Tables

**Table 2:** Comparison of the MFI test results of virgin PLA (vPLA), recycled PETG (rPETG), rPET90/rHDPE10, and rPET90/rHDPE10/SEBS10 obtained with the commercial and OS MFI devices considering the different feedstock types and recycled contents.

Material	Recycled Content	Feedstock Type	Preheat Time (s)		Temperature (°C)	MFI (g/10 min)	
			Commercial	OS		Commercial	OS
vPLA	0 wt.%	Thermoplastic, Pellets	300	600	190	6.0 ± 0.8	5.1 ± 0.3
rPETG	100 wt.%	Thermoplastic, Flakes			240	14.3 ± 1.5	13.6 ± 2.7
					230	9.0 ± 0.6	6.9 ± 0.9
					220	6.5 ± 0.8	4.2 ± 0.5
rPET90/rHDPE10	100 wt.%	Thermoplastic blend, Flakes			255	34.5 ± 4.3	18.3 ± 6.0
rPET90/rHDPE10/SEBS10	100 wt.%	Thermoplastic blend, Flakes			255	31.1 ± 4.3	36.9 ± 15.4

**Table 3:** One-way ANOVA analysis of virgin PLA (vPLA), recycled PETG (rPETG), rPET90/rHDPE10, and rPET90/rHDPE10/SEBS10 obtained with the OS MFI device considering the different samples (SS: sum of squares; df: degrees of freedom; MS: mean square; F-ratio: value of the test statistic; *p*: value of probability).

Material	Temperature (°C)	Variation Sources	SS	df	MS	F-Ratio (and F-Crit)	*p*
vPLA	190	Samples (between groups)	0.0797	3	0.0267	11.6355 (2.7249)	0.000002343 (≤0.05)
		Time (within groups)	0.1735	76	0.0023		
		Total	0.2532	79	-		
rPETG	240	Samples (between groups)	0.2227	2	0.1113	4.2468 (3.1682)	0.0193667916 (≤0.05)
		Time (within groups)	1.4164	54	0.0262		
		Total	1.6392	56	-		
	230	Samples (between groups)	0.1069	2	0.0534	13.7586 (3.1682)	0.0000148214 (≤0.05)
		Time (within groups)	0.2099	54	0.0038		
		Total	0.3169	56	-		
	220	Samples (between groups)	0.0259	2	0.0129	28.8799 (3.1588)	0.0000000022 (≤0.05)
		Time (within groups)	0.0256	57	0.0004		
		Total	0.0515	59	-		
rPET90/rHDPE10	255	Samples (between groups)	0.0248	1	0.0248	0.6548 (4.3009)	0.4270409692 (>0.05)
		Time (within groups)	0.8342	22	0.0379		
		Total	0.8590	23	-		
rPET90/rHDPE10/SEBS10	255	Samples (between groups)	0.5035	1	0.5035	2.0669 (4.7472)	0.1760829442 (>0.05)
		Time (within groups)	2.9231	12	0.2435		
		Total	3.4266	13	-		

**Table 4:** One-way ANOVA analysis of virgin PLA (vPLA), recycled PETG (rPETG), rPET90/rHDPE10, and rPET90/rHDPE10/SEBS10 obtained with the OS MFI device considering the measurement time interval (SS: sum of squares; df: degrees of freedom; MS: mean square; F-ratio: value of the test statistic; *p*: value of probability).

Material	Temperature (°C)	Variation Sources	SS	df	MS	F-Ratio (and F Crit)	*p*
vPLA	190	Time (between groups)	0.1342	19	0.0071	3.5616 (1.7625)	0.0000883299 (≤0.05)
		Samples (among groups)	0.1190	60	0.0020		
		Total	0.2532	79	-		
rPETG	240	Time (between groups)	0.5173	18	0.0287	0.9735 (1.8826)	0.5068153904 (>0.05)
		Samples (among groups)	1.1219	38	0.0295		
		Total	1.6392	56	-		
	230	Time (between groups)	0.0619	18	0.0034	0.5122 (1.8826)	0.9352043052 (>0.05)
		Samples (among groups)	0.2250	38	0.0067		
		Total	0.3169	56	-		
	220	Time (between groups)	0.0154	19	0.0008	0.8999 (1.8529)	0.5857087660 (>0.05)
		Samples (among groups)	0.0361	40	0.0009		
		Total	0.0516	59	-		
rPET90/rHDPE10	255	Time (between groups)	0.5010	11	0.0455	1.5267 (2.7173)	0.2389763194 (>0.05)
		Samples (among groups)	0.3580	12	0.0298		
		Total	0.8591	23	-		
rPET90/rHDPE10/SEBS10	255	Time (between groups)	0.9504	6	0.1584	0.4478 (3.8660)	0.8265373010 (>0.05)
		Samples (among groups)	2.4762	7	0.3537		
		Total	3.4266	13	-		

**Table 5:** Two-way ANOVA analysis of recycled PETG (rPETG) tested at different temperatures, i.e., 240 °C, 230 °C, and 220 °C, by using the OS MFI device (SS: sum of squares; df: degrees of freedom; MS: mean square; F-ratio: value of the test statistic; *p*: value of probability).

Material	Temperature (°C)	Variation Sources	SS	df	MS	F-Ratio (and F Crit)	*p*
rPETG	240, 230 and 220	Temperature (samples)	0.2524	2	0.1262	10.1872 (3.0758)	0.0000850201 (≤0.05)
		Time (columns)	0.2157	18	0.0112	0.9670 (1.6950)	0.5020522235 (>0.05)
		Interaction	0.3780	36	0.0105	0.8472 (1.5207)	0.7105052310 (>0.05)
		Within	1.4128	114	0.0124	-	-
		Total	2.2590	170	-	-	-

**Figure A1:**
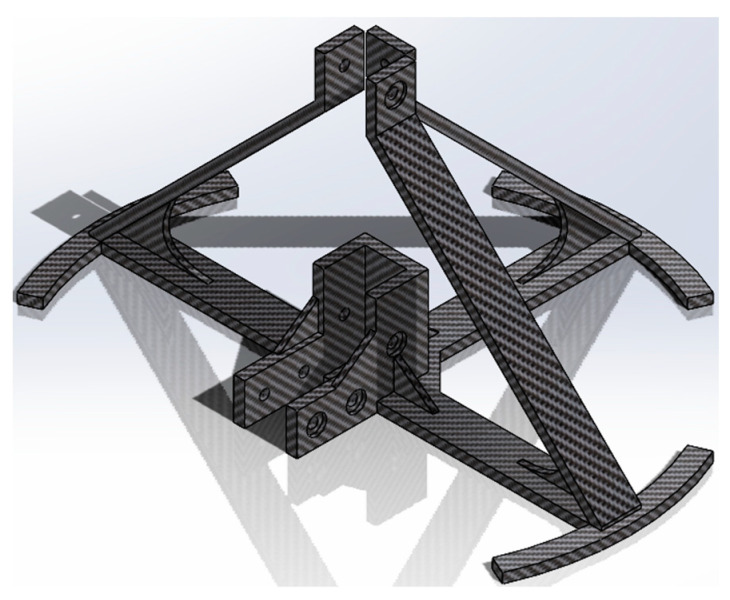
Part 1: Stand.

**Figure A2:**
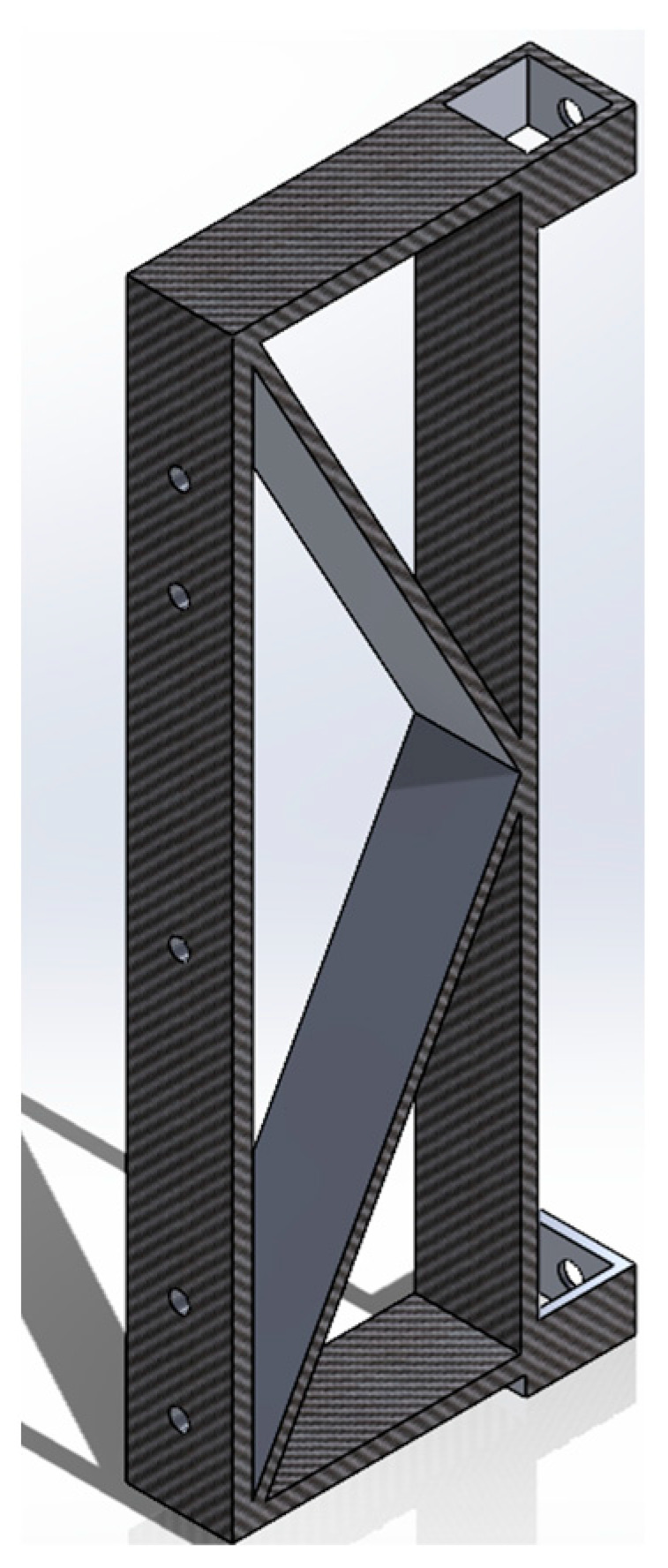
Part 2: linear support.

**Figure A3:**
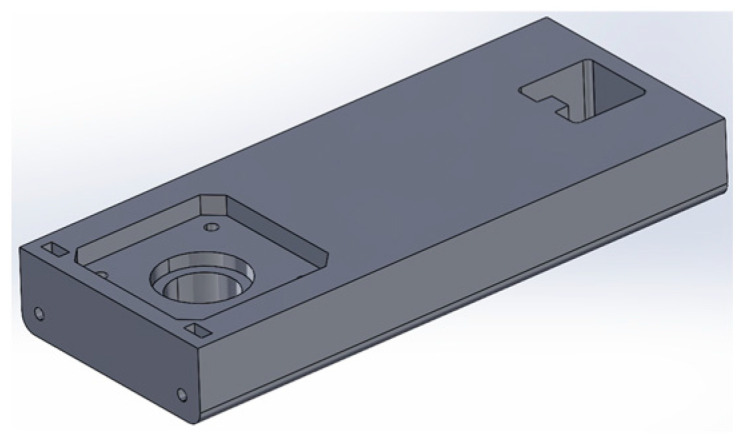
Part 3: motor mount.

**Figure A4:**
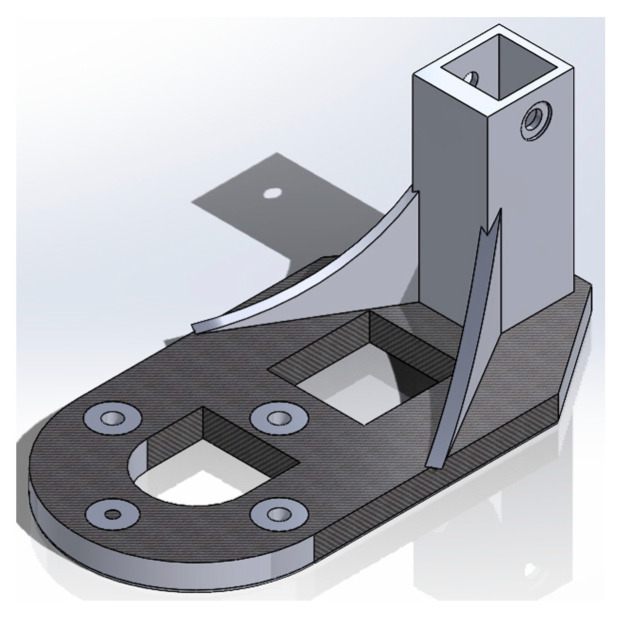
Part 4: flange support.

**Figure A5:**
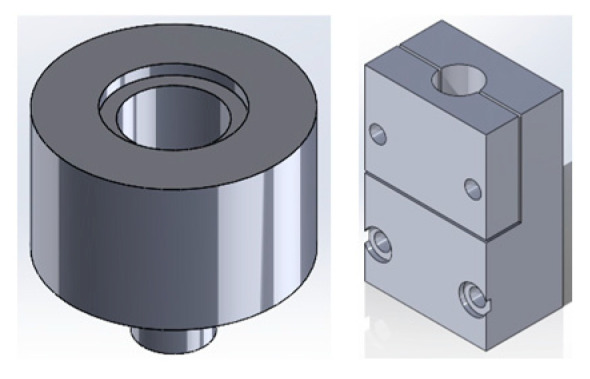
Part 5: loadcell fit (**left**). Part 6: shaft loadcell coupler (**right**).

**Figure A6:**
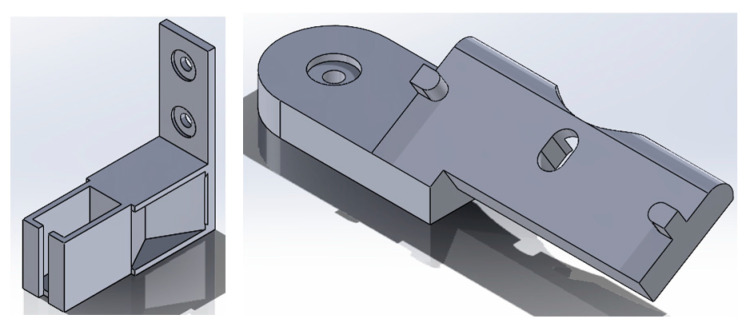
Part 7: cutter connector (**left**). Part 8: blade fix (**right**).

**Figure A7:**
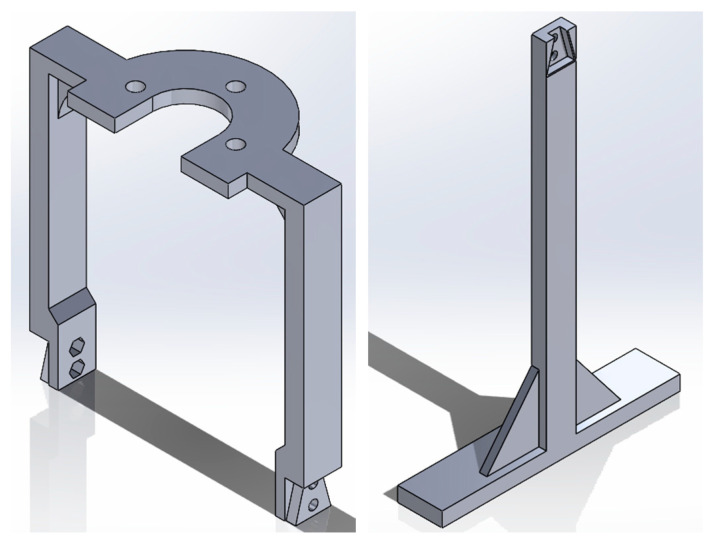
Part 9: extra frame (**left**). Part 10: extra frame b (**right**).

**Figure A8:**
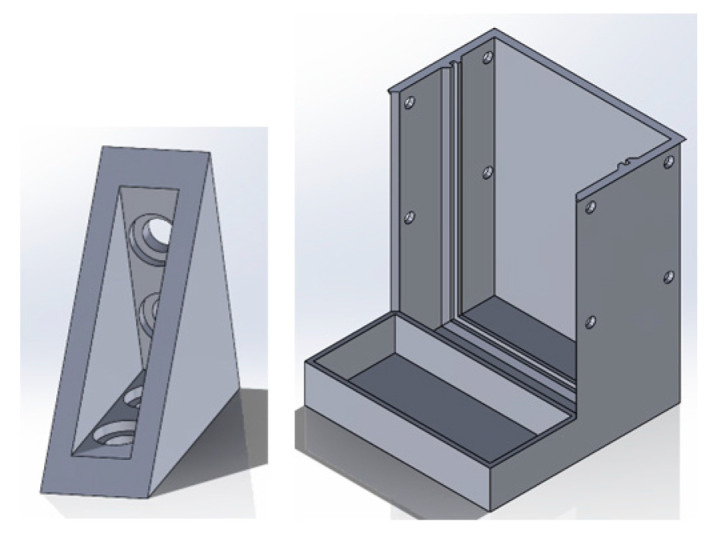
Part 11: foot support (**left**). Part 12: Housing (**right**).

**Figure A9:**
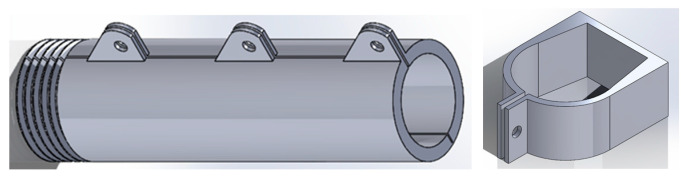
Part 13: insulation housing (**left**). Part 14: cap band (**right**).

**Figure A10:**
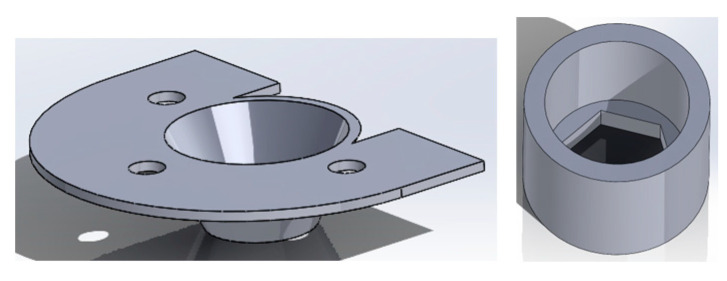
Part 15: piston guide (**left**). Part 16: piston tip guide (**right**).

**Figure A11:**
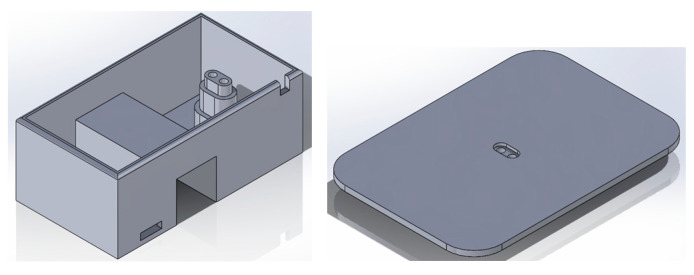
Part 17: scale housing (**left**). Part 18: scale plate (**right**).

**Figure A12:**
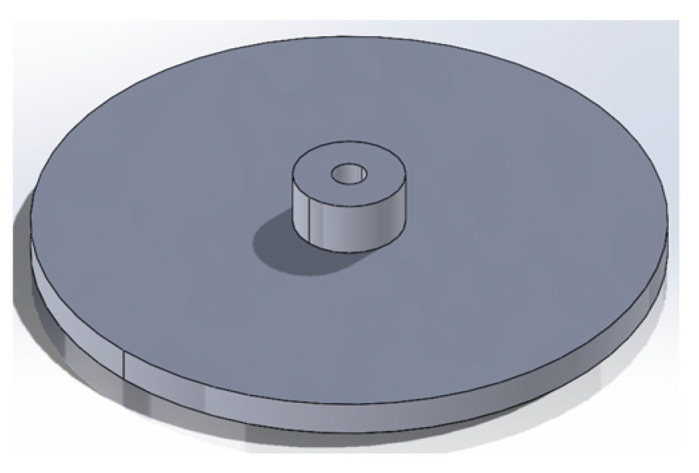
Part 19: calibration part.

**Figure A13:**
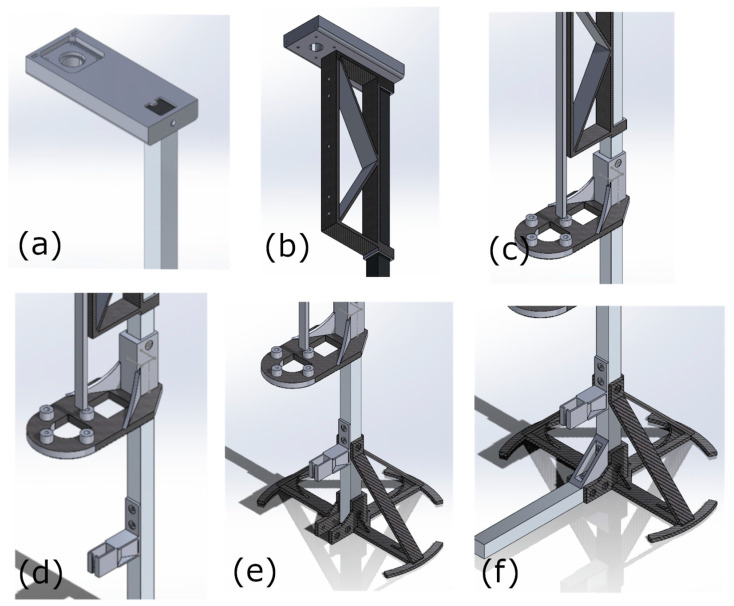
Steps of the frame assembly: (**a**) Motor mount assembly; (**b**) linear support assembly; (**c**) flange assembly; (**d**) cutter assembly; (**e**) stand assembly; and (**f**) foot support assembly.

**Figure A14:**
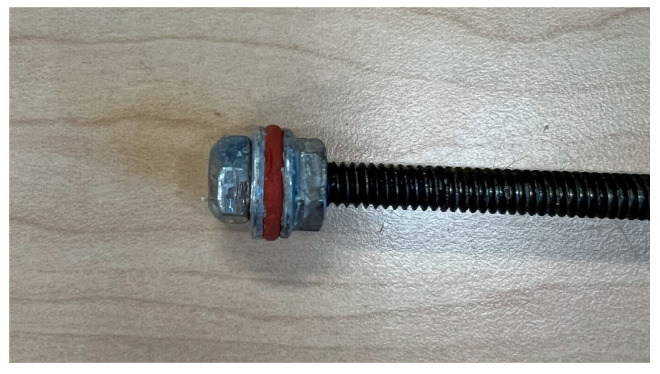
Steps of the piston assembly: piston tip assembly.

**Figure A15:**
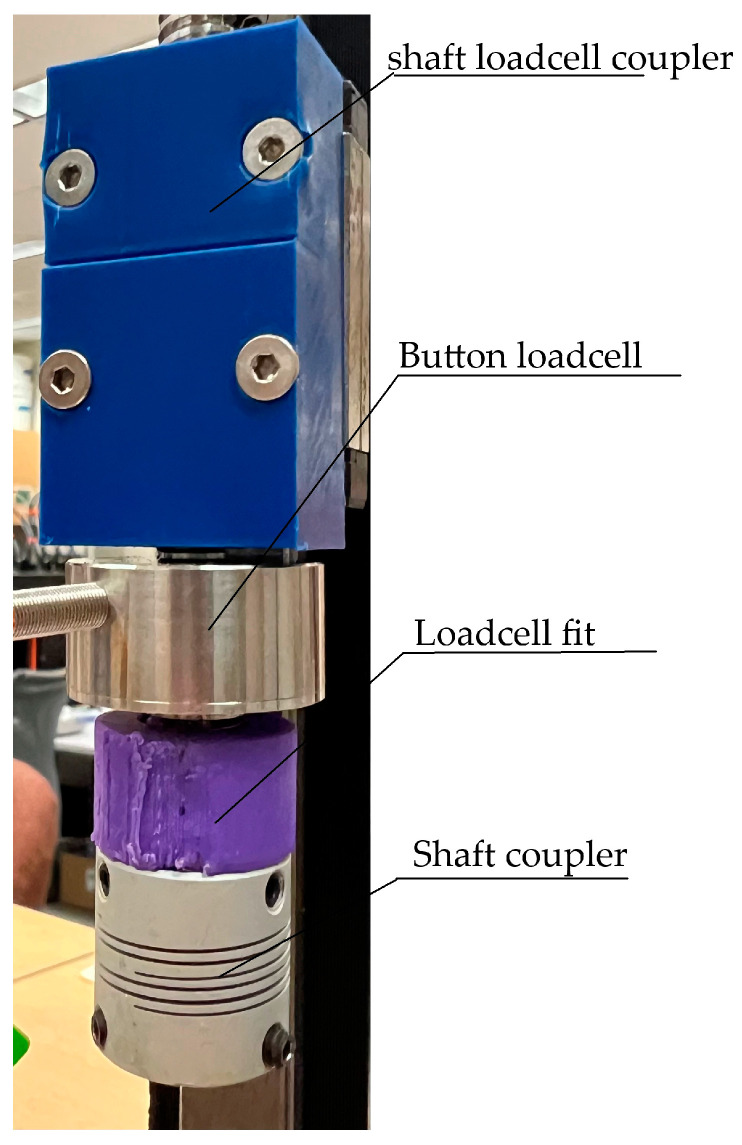
Steps of the piston assembly: coupler connection assembly.

**Figure A16:**
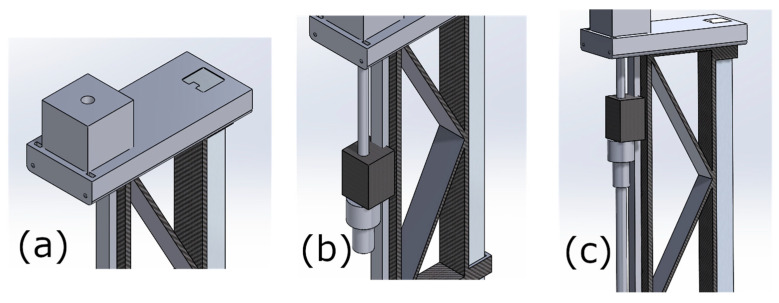
Steps of the piston assembly: (**a**) linear rail assembly; (**b**) loadcell assembly; and (**c**) shaft coupler assembly.

**Figure A17:**
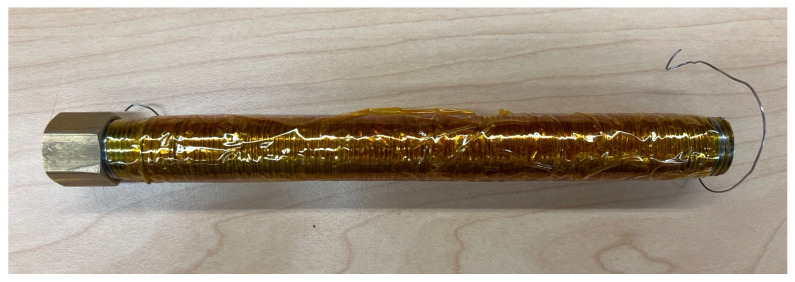
Steps of the heating pipe assembly.

**Figure A18:**
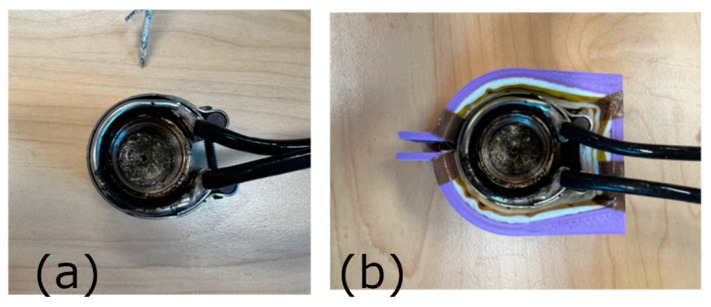
Steps of the cap assembly: (**a**) band heater tightening; (**b**) insulation cap assembly.

**Figure A19:**
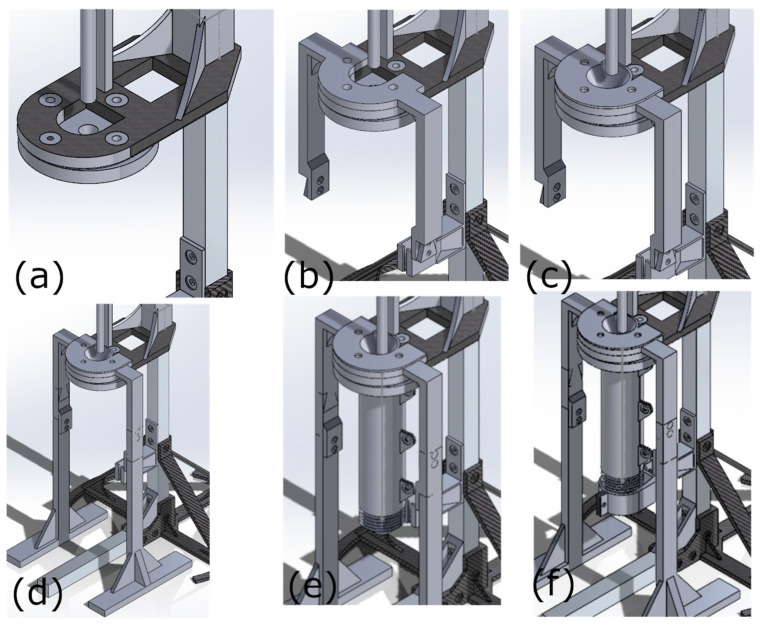
Steps of the heating pipe and cap ssembling: (**a**) flange assembly; (**b**–**d**) extra support assembly; (**e**) pipe assembly; and (**f**) cutting setup adjustment.

**Figure A20:**
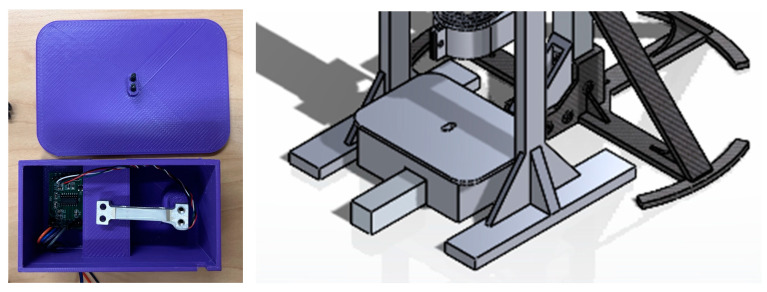
Steps of the scale assembly: HX711 board assembly (**left**)**;** and load cell assembly (**right**).

**Figure A21:**
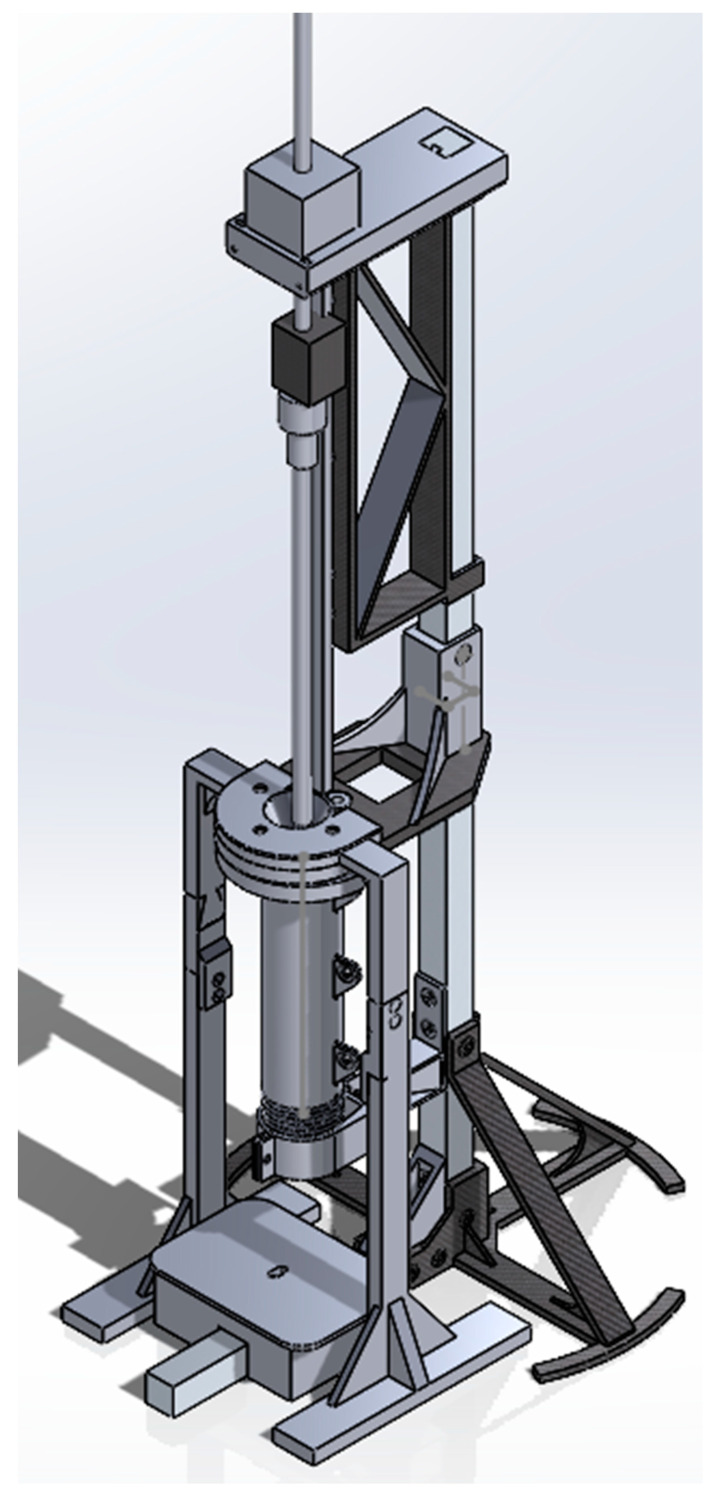
MFI device assembly after the different parts’ assembly.

**Figure A22:**
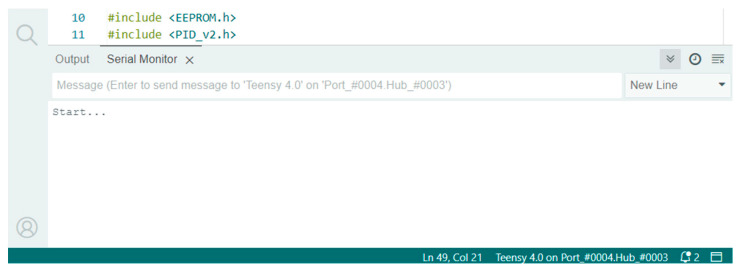
Operation instructions: using the serial monitor in Arduino IDE.

**Figure A23:**
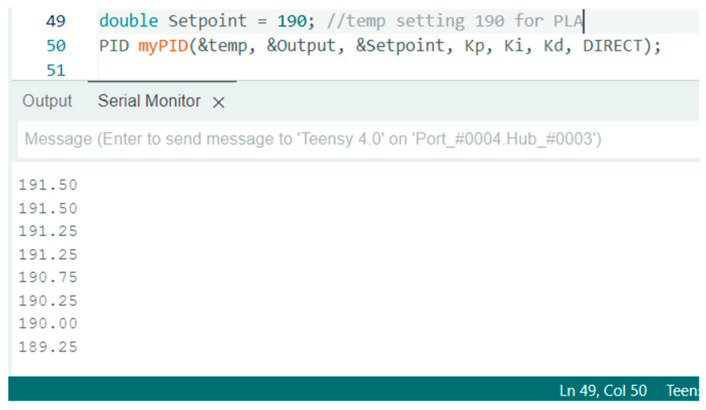
Operation instructions: setting the temperatures for the tests.

**Figure A24:**
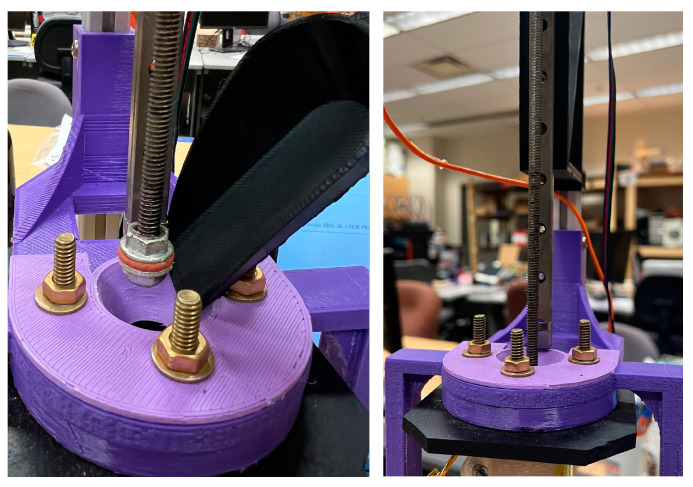
Operation instructions: moving the piston after the preheating procedure (**left**) and reaching the pressure set point to start the testing (**right**).

## Data Availability

The original data presented in the study are openly available in OSF repository at [https://osf.io/68hbj/ (accessed on 29 November 2024)].

## Appendix A

The OS MFI device parts are listed here below. The overall cost for its building is around CAD 318.15 before taxes, according to the specific spare parts or 3D printing filament suppliers.

### Appendix A.1. Make Parts

The bill of materials (BOM) for the make parts is shown in Table A1. The full list with links to commercially available components can be found at the OSF repository [81]. All files are licensed under GNU GPL v3.

**Table A1:** BOM of the make parts and files for the OS MFI device (hardware).

No.	Design File Name	File Type	Technology	Material
1	Stand	STL&STEP	FFF 3D printing	PETG
2	Linear support	STL&STEP	FFF 3D printing	PETG
3	Motor mount	STL&STEP	FFF 3D printing	PETG
4	Flange support	STL&STEP	FFF 3D printing	PETG
5	Loadcell fit	STL&STEP	FFF 3D printing	PETG
6	Shaft loadcell coupler	STL&STEP	FFF 3D printing	PETG
7	Cutter connector	STL&STEP	FFF 3D printing	PETG
8	Blade fix	STL&STEP	FFF 3D printing	PETG
9	Extra frame	STL&STEP	FFF 3D printing	PETG
10	Extra frame b	STL&STEP	FFF 3D printing	PETG
11	Foot support	STL&STEP	FFF 3D printing	PETG
12	Housing	STL&STEP	FFF 3D printing	PETG
13	Insulation housing	STL&STEP	FFF 3D printing	PC
14	Cap band	STL&STEP	FFF 3D printing	PC
15	Piston guide	STL&STEP	FFF 3D printing	PETG
16	Piston tip guide	STL&STEP	FFF 3D printing	PETG
17	Scale housing	STL&STEP	FFF 3D printing	PETG
18	Scale plate	STL&STEP	FFF 3D printing	PETG
19	Block	STL&STEP	FFF 3D printing	PETG
20	Calibration part	STL&STEP	FFF 3D printing	PETG
21	PCB	zip		//
22	MFI	ino		//

The make parts are listed hereinafter with a short description and a rendering preview of the component.

Part 1 (File 1). Stand: this part is the basement of the whole frame, which can hold the two aluminum profiles 90 degrees, making the whole structure vertical (Figure A1).

Part 2 (File 2). Linear support: this part can support the linear rail and the motor mount part, and mount the rail to the aluminum extrusion profile (Figure A2).

Part 3 (File 3). Motor mount: this part is meant to support and mount the motor on the aluminum profile (Figure A3).

Part 4 (File 4). Flange support: this L-shaped part supports the heated barrel with two external ribs to increase strength (Figure A4).

Part 5 (File 5). Loadcell fit: this part is used to help connect the shaft coupler and the load cell (Figure A5, left).

Part 6 (File 6). Shaft loadcell coupler: this rectangular part with top and bottom holes connects the load to the piston. There are four blot holes, which correspond to the slide block of the linear rail. The top part is separate, which can help it grip the motor shaft (Figure A5, right).

Part 7 (File 7). Cutter connector: this part mounts the servo motor on the aluminum extrusion profile (Figure A6, left).

Part 8 (File 8). Blade fix: this part attaches the blade to the servo motor (Figure A6, right).

Part 9 (File 9). Extra frame: this part provides extra support for the flange support so that it will not deform when the piston presses down (Figure A7, left).

Part 10 (File 10). Extra frame b: this part is the bottom part of the extra frame (Figure A7, right).

Part 11 (File 11). Foot support: this part is the triangle part that goes between two aluminum extrusion profiles, so that they can keep stand vertically (Figure A8, left).

Part 12 (File 12). Housing: this part is the shell of all electronic components (Figure A8, right).

Part 13 (File 13). Insulation housing: this part is the housing for the insulation layer of the heating pipe, which helps hold insulation in place (Figure A9, left).

Part 14 (File 14). Cap band: this part is the band-shaped insulation housing for the band heater. The insulation layer is attached to this part so that the insulation can be taken off easily (Figure A9, right).

Part 15 (File 15). Piston guide: this part helps guide the piston inserted into the pipe. It also works as a fuel when adding materials (Figure A10, left).

Part 16 (File 16). Piston tip guide: this small cap for the piston tip helps keep all the components of the piston tip aligned with the same center point (Figure A10, right).

Part 17 (File 17). Scale housing: this part is the housing for all the scale components. The scale is apart from the main body of the MFI included device to avoid vibration (Figure A11, left).

Part 18 (File 18). Scale plate: this part is a plate that can hold extrusions of the MFI (Figure A11, right).

Part 19 (File 20). Calibration part: this is a calibration part to be 3D-printed for the button load cell. Screw the thread on the load cell to the corresponding hole on this part (Figure A12).

### Appendix A.2. Electronic Parts and Buy/Mechanical Components

The BOM for the electronic components is shown in Table A2, whereas the BOM of the buy/mechanical components is visible in Table A3. The full list with links to commercially available components can be found at the OSF repository [81].

**Table A2:** BOM of electronic components.

No.	Name	Model Number	Quantity	Cost per Unit (CAD)	Source
1	PCB		1	10.00	JLCPCB
2	Control board	Teensy board 4.0	1	23.80	Teensy.com
3	Motor driver	TMC2130	1	10.0	Amazon
4	Amplifier	HX711	2	6.00	Amazon
5	ADC	MAX31855KASA	1	12.66	Digikey
6	LDO regulator	AMS1117	1	0.85	Digikey
7	Force sensor	DYMH-103	1	70.42	AliExpress
8	Loadcell	100 g	1	10.79	Amazon
9	SMD Resistor	10 k	2	0.15	Digikey
10	Capacitors c1	100 uF	1	0.48	Digikey
11	Capacitors c2	22 uF	1	0.21	Digikey
12	Capacitors c3	0.01 uF	1	0.14	Digikey
13	Capacitors c4	10 uF	1	0.14	Digikey
14	Ferrite Beads	FERRITE BEAD 120 OHM 0603 1LN	2	0.18	Digikey
15	Thermocouple	240-080	1	15.80	Digikey
16	Connecters		8	0.10	Amazon
17	Motor	Nema 17 Non-captive Linear stepper motor	1	25.84	AliExpress
18	Servomotor		1	2.90	Amazon
19	Power supply	110 v AC to 12 V DC	1	22.88	Amazon
20	Relay module	12V	1	1.90	Amazon
21	Wires	Jump wires	10	0.10	Amazon
22	High temperature-resist wires	Awclub Mica	0.164 ft	0.75/ft	Amazon
23	High temperature-resist connectors	O Type and U Type	8	0.05	Amazon
24	Nichrome wire	50′ nichrome 80 wire	1	9.47	Master wire supply
25	Band heater	12 V 40 W	1	20.00	Filastruder

**Table A3:** BOM of buy/mechanical components.

No.	Name	Model Number	Quantity	Cost per Unit (CAD)	Source	Material
1	Bolts b1	M6-5 mm	19	0.10	Amazon	Steel
2	Bolts b2	M3-20 mm	8	0.10	Amazon	Steel
3	Bolts b3	M6-40 mm	3	0.10	Amazon	Steel
4	Bolts b4	M2-8 mm	4	0.10	Amazon	Steel
5	Bolts b5	M3-15 mm	3	0.10	Amazon	Steel
6	Nuts n1	M6	3	0.24	Amazon	Steel
7	Nuts n2	M3	3	0.10	Amazon	Steel
8	Nuts n3	M2	2	0.09	Amazon	Steel
9	T-nuts t1	M6, 20 × 20	19	0.20	Amazon	Steel
10	Serrated Flange Lock Nut	¼ inch	1	0.80	Amazon	Steel
11	Self-lock nut	¼ inch	1		Amazon	Steel
12	Washers w1	M6	4	0.10	Amazon	Steel
13	Washer w2	M3	2	0.10	Amazon	Steel
14	Heat tube	½ inches	1	20.00	McMaster-Carr	Steel
15	cap	½ inches, hex	1	5.00	McMaster-Carr	Copper
16	Aluminum profile a1	20 × 20	75 mm	0.013/mm	McMaster-Carr	Aluminum
17	Aluminum profile a2	20 × 20	30 mm	0.013/mm	McMaster-Carr	Aluminum
18	Linear rail	350 mm	2	0.18	Amazon	Steel
19	Blade	240-080	1	15.80	McMaster-Carr	Steel
20	Piston rod	¼ inches, 12 inches length	2	1.50	Home Depot	Steel
21	O-ring	OD5/8	1	0.26	McMaster-Carr	Silicon
22	Capton tape		1	2.90	Amazon	Mixed
23	Shaft Coupler	¼ inch	1	3.00	Amazon	Steel
24	Insert Nuts	3 mm	2	1.90	McMaster-Carr	Steel
25	Insulation	heat resistance cotton	1.50 mm^2^	0.08/inch^2^	McMaster-Carr	Fiberglass

## Appendix B

**Build Instructions**

### Appendix B.1. Mechanics

#### Appendix B.1.1. Step 1. Frame Assembly

The assembly procedure is resumed in Figure A13 and described hereinafter:

- Insert the T-nut into the slot of the 1-m aluminum profile, and assemble the 3D-printed part motor mount on the top of the aluminum extrusion profile (Figure A13a);
- Insert the same aluminum profile into the two holes of linear support. Push the linear support until it touches the motor support, then secure it to the aluminum extrusion profile with bolts and T-nuts. Attach the linear rail to the linear support with fasteners using the corresponding holes (Figure A13b);
- Insert the aluminum profile into the 3D-printed flange support and fix the position with bolts and T-nuts. The position depends on the length of the linear rail (Figure A13c);
- Put the 3D-printed part of the cutter connector in the right position and fix it with bolts and nuts. The position depends on the length of the heating pipe. It should allow the cutter to cut off the extrusions (Figure A13d);
- Insert the aluminum extrusion profile into the 3D-printed stand and fix it with fasteners (Figure A13e);
- Attach another aluminum extrusion profile to the stand and fix the position with fasteners. Then assemble the 3D-printed foot support and make sure the two aluminum extrusion profiles are vertical (Figure A13f).

#### Appendix B.1.2. Step 2. Piston Assembly

The assembly procedure is resumed in Figure A14, Figure A15 and Figure A16, described hereinafter:

- Screw the ¼ inch serrated flange lock nut to the ¼-inch threaded rod and stop at 15 mm away from the tip. Put an O-ring on top of the nut, then put a washer on top of it. Screw another self-lock nut, cover it with a piston guide, and ensure that all pieces have the same center point. Use the 3D-printed piston tip guide to make sure all the components are centered and not too tight. If the screw is too tight, the O-ring in the middle will deform, which makes it harder to insert into the heating pipe (Figure A14).
- Assemble the motor on the 3D-printed motor support. Connect the shaft of the motor to the shaft loadcell coupler (Figure A15);
- Screw the loadcell fit into the shaft loadcell coupler, then install it on the slide block of the linear rail (Figure A16a);
- Screw the insert nut into the 3D-printed loadcell fit, connect the loadcell fit and the button loadcell, then screw the button loadcell to the shaft loadcell coupler (Figure A16b);
- Connect the shaft coupler to the loadcell fit. Connect the piston rod with the shaft coupler (Figure A16c).

#### Appendix B.1.3. Step 3. Heating Pipe and Cap Assembly

The assembly procedure is resumed in Figure A17, Figure A18 and Figure A19, described hereinafter:

- Cover the whole pipe with high-temperature-resistant tape (Kapton Tape). Wind nichrome wire around the iron pipe as required, ensuring the nichrome wire has sufficient resistance to at least meet the maximum current requirements of the relay. The nichrome wires must not touch each other to prevent short-circuiting or excessive heat generation at the contact points. After winding, wrap high-temperature tape around the outside of the resistance wire to achieve insulation (Figure A17);
- Tape the thermocouple to the outside of the resistance wire;
- Cover the pipe with heat insulation material, such as fiberglass. Cover the fiberglass layer with Kapton tape so that it can keep on the pipe;
- Cover the insulation layer with 3D-printed housing (insulation housing);
- Drill a 2 mm hole in the center of the cap. Insert the cap in the band heater (Figure A18a);
- Tighten the screw on the band heater. Cover the band heater with a 3D-printed insulation cap band (Figure A18b);
- Screw the cap on one end of the pipe;
- Attach the flange and insulation pad to the 3D-printed part of the flange support with bolts and nuts. The insulation pad should be between the flange and the flange support (Figure A19a);
- Fix the extra frame and piston guide with the same bolts and nuts as the last step (Figure A19b–d);
- Screw the pipe to the flange (Figure A19e);
- Adjust the position of the blade. It should touch the bottom of the cap (Figure A19f).

#### Appendix B.1.4. Step 4. Digital Scale Assembly

The assembly procedure is resumed in Figure A20 and described hereinafter:

- Connect wires on the HX711 board;
- Assemble the scale platform on the load cell;
- Fix the HX711 board on the related slot in the scale housing (Figure A20, left);
- Assemble the load cell on the scale housing (Figure A20, right);
- Pull jump wires out and connect the other end to the main board.

The assembly of the MFI is finished (Figure A21), and the device is ready for electronic wiring before the first calibrations.

### Appendix B.2. Electronic Wiring

- The electronic wiring procedure is resumed: Connect a high-temperature-resistant connector to one end of the high-temperature-resistant wire and connect it to the nichrome wire.
- Follow the instructions in the schematic in the Open Science Framework [81].

***Operation and cleaning instructions***

### Appendix B.3. Operational Instructions

A first preparation step is required before using the device for testing. In detail, humidity-sensitive materials like PETG and PLA should be dried before measurement. Get a specific weight of the material, such as 10 g, and dry it. The drying process should follow the standard profile. The operation instructions are resumed in Figure A22, Figure A23 and Figure A24, described hereinafter:

1. Connect the Teensy board with the laptop. Plug in the power supply (Figure A22);
2. Open the .io file with Arduino IDE and download all the libraries. Setup parameters are needed, and the new firmware is uploaded to the Teensy board. More detailed instructions are in the code file;
3. Open the Serial Monitor in Arduino IDE, baud rate 115200. “start” should show up on the Serial monitor (Figure A22);
4. Input the start command monitor “a” in Message blank. The temperature will increase, and the piston will start to go down automatically after the heating-up procedure ends;
5. Fill in the samples when the temperature reaches the set point (Figure A23);
6. Bring down the piston using the command “d”. The piston will start to go down automatically after the heating-up procedure ends (Figure A24, left);
7. Wait for the pressure to get to the set point. Record the readings after the cutter cuts off samples (Figure A24, right);
8. Clean the barrel and the cap after the measurement is finished.

Data processing should be performed by getting at least 20 datasets, where the first X datasets should be abandoned, e.g., for thermal stabilization and complete melting of the material sample (preheating). This fact is due to the initial temperature’s instability in the heated barrel and die. Only the last X sets of data should be considered for MFI measurement to achieve accurate results.

Before each test, it is advisable to check the extrudability of the material through the die, ensuring the absence of clogging or jams that can affect the flowability of the extrudate samples, e.g., under-extrusion and lower MFI values. Cleaning the die after each test or batch of samples can help ensure consistency among the different measurements. This procedure can be done with a needle fitting the nozzle diameter. Tests should be performed at room temperature, ensuring consistent room conditions for each measurement, e.g., temperature and humidity. Particular attention is needed when testing particle-filled materials, as they can lead to increased wear of the heated barrel and die, decreasing their accuracy and requiring fine-tuning to assess the actual extruded mass during the measurements or part replacement.

### Appendix B.4. Cleaning Instructions

The cleaning instructions are described in detail in the following:

1. Go into cleaning mode by inputting the command in the Arduino IDE serial monitor to keep the temperature;
2. Dissemble the insulation band, unplug the band heater, and unscrew the cap with a clamp or heat-resistant gloves;
3. Plug the band heater back and clean the cap with manual cleaning tools, such as a copper brush, nozzle needles, or pliers. Cleaning agents should be used after manual cleaning to remove the small residues from the measurements, and should be used when the parts are cooled down, selecting them according to the equipment and sample material, e.g., isopropyl alcohol;
4. Pull out the piston and clean the piston tip with manual cleaning tools and cleaning agents, if needed;
5. Clean the tube with the piston and cover the piston tip with a cotton rug or robust paper;
6. Screw the cap back after the cleaning process.
